# A Biomarker-based Biological Age in UK Biobank: Composition and Prediction of Mortality and Hospital Admissions

**DOI:** 10.1093/gerona/glab069

**Published:** 2021-03-06

**Authors:** Mei Sum Chan, Matthew Arnold, Alison Offer, Imen Hammami, Marion Mafham, Jane Armitage, Rafael Perera, Sarah Parish

**Affiliations:** 1 Nuffield Department of Population Health, University of Oxford, UK; 2 British Heart Foundation Cardiovascular Epidemiology Unit, Department of Public Health and Primary Care, University of Cambridge, UK; 3 MRC Population Health Research Unit, University of Oxford, UK; 4 Nuffield Department of Primary Health Care Sciences, University of Oxford, UK

**Keywords:** Epidemiology, Outcomes, Preventative health care, Risk factors

## Abstract

**Background:**

Chronological age is the strongest risk factor for most chronic diseases. Developing a biomarker-based age and understanding its most important contributing biomarkers may shed light on the effects of age on later-life health and inform opportunities for disease prevention.

**Methods:**

A subpopulation of 141 254 individuals healthy at baseline were studied, from among 480 019 UK Biobank participants aged 40–70 recruited in 2006–2010, and followed up for 6–12 years via linked death and secondary care records. Principal components of 72 biomarkers measured at baseline were characterized and used to construct sex-specific composite biomarker ages using the Klemera Doubal method, which derived a weighted sum of biomarker principal components based on their linear associations with chronological age. Biomarker importance in the biomarker ages was assessed by the proportion of the variation in the biomarker ages that each explained. The proportions of the overall biomarker and chronological age effects on mortality and age-related hospital admissions explained by the biomarker ages were compared using likelihoods in Cox proportional hazard models.

**Results:**

Reduced lung function, kidney function, reaction time, insulin-like growth factor 1, hand grip strength, and higher blood pressure were key contributors to the derived biomarker age in both men and women. The biomarker ages accounted for >65% and >84% of the apparent effect of age on mortality and hospital admissions for the healthy and whole populations, respectively, and significantly improved prediction of mortality (*p* < .001) and hospital admissions (*p* < 1 × 10^−10^) over chronological age alone.

**Conclusions:**

This study suggests that a broader, multisystem approach to research and prevention of diseases of aging warrants consideration.

Chronological age is the strongest risk factor for most chronic diseases that limit healthy life span, but individuals may age biologically at different rates (1), characterized by differential rates of disease accumulation and frailty onset. Understanding the contributors to biological aging could lead to opportunities for early prevention of later-life disease (2) and limiting the disease burden associated with aging populations.

A commonly used approach to identifying risk of accelerated aging and reduced life span is to form a risk score by regressing mortality on risk factors (2,3). However, this tends to identify people who have known health conditions (2). It would be particularly advantageous to be able to identify accelerated aging in apparently healthy people for primary prevention of diseases of aging (4). Another approach involves estimating a person’s biological age from the age that their biomarker profile typically reflects. In a review of estimation methods for biological ages (5), studies that compared different estimation methods (6–8) favored the Klemera Doubal method (KDM), which derives a weighted sum of biomarkers based on the strengths of their associations with chronological age (9). Additionally, in a more recent study in an older Singaporean population, biological age estimated using KDM was found to be more predictive of mortality and frailty than biological ages estimated using machine learning methods (8). When estimating a KDM biological age, chronological age may or may not be treated as a constituent “biomarker”; if it is not a constituent “biomarker,” the predictive value of the derived biological age for health outcomes can be compared with that of chronological age. KDM can be implemented using principal components of biomarkers instead of individual biomarkers (6), avoiding the problem of overweighting toward multiple correlated biomarkers.

The UK Biobank is a richly phenotyped resource with 0.5 million participants (10) that provides an unrivalled opportunity to investigate earlier stages of aging through biological, lifestyle, and environmental factors easily measured at scale, compared to previous clinical biomarker-based studies of biological aging typically based on 100–10 000 participants with panels of fewer than 30 biomarkers (5). A substantial middle-aged and apparently healthy subpopulation of the UK Biobank can be identified, to assess the prognostic capability of a biomarker age for subsequent health and to reduce reverse causality from prior health or medication use affecting biomarker levels.

This study aims to focus on healthy individuals and (i) estimate sex-specific biomarker ages in the UK Biobank using the KDM, (ii) identify the main biomarker determinants of the biomarker ages, and (iii) investigate the relationship between the biomarker age and chronological age in the prediction of mortality from chronic diseases and age-related hospital admissions.

## Method

### Study Population

The UK Biobank recruited 0.5 million participants across the United Kingdom aged 40–70 for baseline assessment in 2006–2010. Information on sociodemographic characteristics, self-reported health behaviors, and medication was recorded. Linkage to Hospital Episode Statistics and national death registries provided prior and prospective information on secondary care outcomes and date and cause of death (Supplementary Appendices 1 and 2) (10). This study was covered by the general ethical approval for UK Biobank studies from the NHS National Research Ethics Service on June 17, 2011 (Ref 11/NW/0382).

After data cleaning (Supplementary Appendix 1 and Supplementary Figure 1), 480 019 participants followed up for 6–12 years via death registry and Hospital Episode Statistics records were included (Supplementary Figure 1). In order to reduce reverse causality, this study focused on the 141 254 people healthy at baseline, who had no chronic disease medications, good health, steady/brisk walk speed, never/ex-smoker (as self-reported); and 0–2 secondary care episodes prior to recruitment, and no prior chronic age-related disease or hip/wrist fracture (in secondary care records) (Supplementary Appendix 1).

### Statistical Analyses

Among 110 physical and biochemical biomarkers commonly measured in clinical settings, 72 biomarkers met quality control standards (Supplementary Appendix 2). Trends of each biomarker with chronological age were visually assessed for linearity (Supplementary Appendix 3A). Principal component analysis with varimax rotation (6) was used to represent the 72 biomarkers as linearly uncorrelated principal components, from which the 51 with eigenvalues >0.33 (more than 1/3 of the average variation described by each biomarker) were taken forward and characterized based on their constituent biomarkers with the largest factor loadings (Supplementary Appendix 3B). The varimax rotation attempts to rotate these components into a simple, easily interpretable structure where only one or a few biomarkers have high loadings in each component, and resulted in many biomarker principal components having only a single biomarker strongly loaded onto them. As would be expected, the principal components for adiposity, lung function, blood pressure, and blood lipids had multiple biomarkers strongly loaded onto them (Supplementary Figure 3).

Biomarker ages were estimated in the healthy population separately for each sex, using the KDM (9) on the 51 principal components, without including chronological age as a biomarker (Supplementary Appendix 3B). The proportion of variation in chronological age attributed to the biomarker age (*R*^2^) was estimated and biomarker principal components were ranked by their importance, measured by the proportion of variance in the biomarker ages that they each explained (Supplementary Appendix 3C).

Two general health outcomes were constructed from Hospital Episode Statistics and death records: death from chronic disease (excluding infectious diseases, pregnancy, congenital malformations, and external causes) (11) and age-related nonfatal hospital admissions (the subset of those types of admission diagnoses in a published hospital frailty risk score (12) that were age-related in the UK Biobank; Supplementary Appendix 2 and Supplementary Table 2). The proportion of the overall biomarker and chronological age effect on hospital admission risk and mortality that was explained by each biomarker age was also estimated, by comparing the log-likelihoods from these Cox models (Supplementary Appendix 3D), and estimating *p* values for the addition of the biomarker age by likelihood ratio tests.

Calibration of the biomarker ages to chronological age was undertaken by plotting the mean biomarker ages for each 2.5-year chronological age group. Risk calibration of biomarker ages with each health outcome was assessed by comparing the Kaplan–Meier survival curves of participants with a biomarker age at least 5 years younger, similar to, and at least 5 years older than their chronological age.

The predictive powers of chronological age and the biomarker age for each health outcome were further characterized by computing Harrell’s C-indices (measures of statistical discrimination similar to the area under the receiver operating curve; Supplementary Appendix 3D) which were calculated both unadjusted and with adjustment for Index of Multiple Deprivation (IMD) 2010 quintile, smoking status, alcohol consumption, and assessment center. As a sensitivity test, prediction of hospital admissions by biomarker age was compared with a benchmark of prediction by a mortality score similar to those proposed by previous studies (2,3), derived using stepwise Cox regression on the 51 biomarker principal components (Supplementary Appendix 3B).

As a sensitivity analysis to investigate whether a smaller (more practical) biomarker panel would suffice, analyses among healthy participants were repeated using the main constituent biomarkers in the 10 most important biomarker principal components in the biomarker ages for each sex. In addition, to aid comparison with previous studies, analyses using the full panel were also undertaken in the whole population.

Guidelines for Transparent Reporting of a multivariable prediction model for Individual Prognosis Or Diagnosis (13) were followed.

## Results

### Study Characteristics

Of the 480 019 participants, 141 254 (29.4%) were in the healthy subpopulation (Table 1). During a median follow-up period of 8.7 years for mortality and 8.0 years for hospital admissions, among healthy participants, 1.7% died from chronic diseases and 16.0% who had not been admitted to hospital with an age-related diagnosis prior to baseline were admitted with such a diagnosis during follow-up (Table 1); the corresponding percentages among the whole population were 3.9% and 23.1%, respectively. Sociodemographic patterns and the proportions of participants healthy at baseline were similar between sexes.

**Table 1.** Participant Characteristics for the Healthy Subpopulation and the Whole UK Biobank Population

	Healthy Subpopulation			Whole Population		
	Persons	Men	Women	Persons	Men	Women
Participants (*n*)	141 254	65 869	75 385	480 019	219 248	260 771
Person-years at risk (millions)	1.2	0.56	0.64	4.12	1.87	2.25
Median age at baseline (years)	56.0	55.7	56.4	58.3	58.8	58.0
Age band at baseline in years (%)						
40–44	12.1	13.9	10.5	10.2	10.4	10.1
45–49	15.9	16.5	15.4	13.1	12.7	13.4
50–54	17.8	17.1	18.4	15.1	14.4	15.8
55–59	19.4	18.6	20.1	18.1	17.5	18.6
60–64	21.9	21.0	22.6	24.3	24.3	24.3
65–70	12.9	12.9	13.0	19.2	20.8	17.9
IMD 2010 quintile (%)						
Q1 (least deprived)	23.9	23.8	24.1	20.0	19.7	20.1
Q2	22.2	22.1	22.3	20.0	19.7	20.3
Q3	20.9	20.7	21.1	20.0	19.8	20.2
Q4	18.7	18.7	18.7	20.0	19.9	20.1
Q5 (most deprived)	14.3	14.7	13.9	20.0	20.9	19.3
Smoker status (%)						
Current	0.0	0.0	0.0	10.5	12.4	8.8
Previous	33.4	35.9	31.2	34.5	38.3	31.3
Never	66.6	64.1	68.8	54.5	48.8	59.4
No answer/missing	0.0	0.0	0.0	0.5	0.5	0.5
Alcohol consumption frequency (%)						
Never	5.5	4.5	6.5	8.0	6.3	9.5
Special occasions only	8.8	5.6	11.6	11.5	7.3	15.0
One to three times a month	10.5	8.6	12.3	11.1	8.9	13.0
Once or twice a week	27.3	27.2	27.4	25.8	25.9	25.7
Three or four times a week	26.8	29.5	24.5	23.1	26.1	20.5
Daily or almost daily	21.0	24.7	17.7	20.3	25.3	16.1
No answer/missing	0.0	0.0	0.0	0.2	0.2	0.2
Health events during follow-up (*n*)						
Deaths from chronic disease	2394	1357	1037	18 799	11 362	7437
Prior age-related hospital admissions	6206	2953	3253	74 811	35 401	39 410
Incident age-related hospital admissions	21 627	10 317	11 310	93 716	43 700	50 016

*Note*: IMD = Index of Multiple Deprivation.

### Biomarker Characteristics

In the healthy subset, the relationships of most candidate biomarkers with chronological age were broadly linear or flat (Supplementary Figure 2). Lung function biomarkers, systolic blood pressure, cystatin C, and reaction time had the strongest linear relationships with age (Supplementary Figure 2). In the whole population, body mass index, low-density lipoprotein cholesterol (LDL-C), and diastolic blood pressure had clear inverse U-shaped relationships with age, but these were attenuated in the healthy subpopulation. A few biomarkers (LDL-C, heel bone density, calcium, alkaline phosphatase, and phosphate) displayed substantially different trends between sexes, supporting the need to model within sex-specific strata (Supplementary Figure 2).

### Biomarker Importance in Biomarker Ages

**Figure 1.**
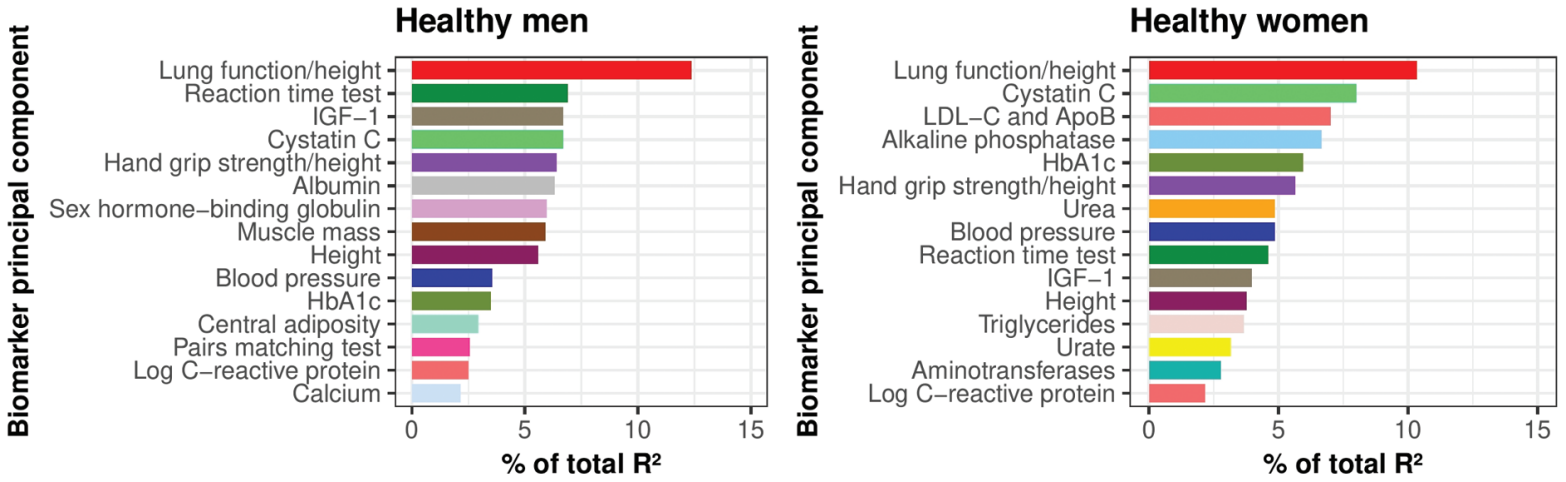
Importance of the top 15 biomarker principal components in the biomarker ages for healthy men and women. The percentage of *R*^2^ denotes the percentage of variation in the biomarker age explained by each biomarker.

The coefficients for the biomarker principal components in the estimated biomarker ages are listed in Supplementary Table 3. The biomarker ages described 44.0% and 51.3% of the variation in chronological age for healthy men and women, respectively. Reduced lung function featured most strongly (Figure 1), describing 12.4% (men) and 10.3% (women) of the variation in the biomarker ages (Supplementary Table 4). Higher cystatin C, slower reaction time, lower insulin-like growth factor 1 (IGF-1), lower hand grip strength, and higher blood pressure also featured strongly for both sexes; while lower albumin, higher sex hormone-binding globulin, and lower muscle mass biomarkers featured strongly for men; and higher levels of alkaline phosphatase, LDL-C and apolipoprotein B, and HbA1c for women. Multiple body systems were represented by these biomarkers (Supplementary Table 1).

### Relationship Between Biological and Chronological Age

The biomarker ages were well calibrated as they matched healthy participants’ chronological ages on average (Supplementary Figure 4). Adding biomarker ages to the prediction models with chronological age statistically significantly improved model fit (for mortality/hospital admissions: *p* < 1 × 10^−10^/*p* < 1 × 10^−10^ in men; *p* < .001/*p* < 1 × 10^−10^ in women) in unadjusted analyses.

**Figure 2.**
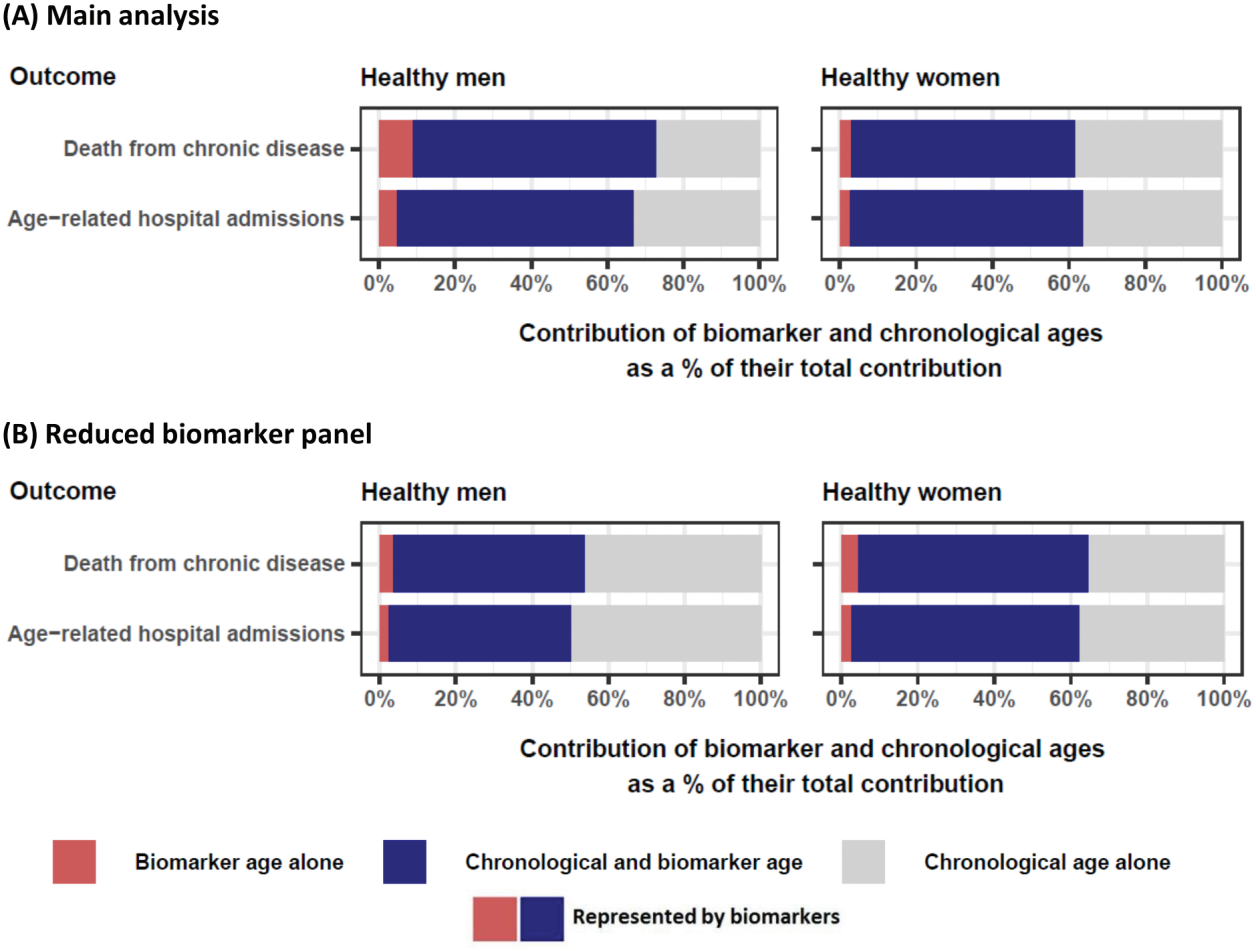
Relative contribution of biomarker ages and chronological age in explaining each health outcome, in the (A) main analysis and when (B) using the reduced biomarker panel, for healthy men and women. The reduced biomarker panel consists of: forced expiratory volume in 1 second/height, forced vital capacity/height, reaction time, insulin growth factor-1, cystatin C, hand grip strength/height, systolic and diastolic blood pressure in both sexes; albumin, sex hormone-binding globulin, fat-free mass, standing height and sitting height in men; and low-density lipoprotein cholesterol, alkaline phosphatase, HbA1c, and urea in women. These were the primary biomarkers that loaded most strongly onto the 10 principal component biomarkers that were most important contributors to biomarker ages for each sex, plus diastolic blood pressure, forced vital capacity, and sitting height because they were strongly loaded onto the same components (rotated factor loading >0.5) and could be measured at the same instance as the primary biomarkers.

More importantly, averaged across sexes, the biomarker ages described 67% and 65% of the overall biomarker age and chronological age effect on mortality and hospital admissions, respectively (Figure 2A) in unadjusted analyses. Constructing the biomarker ages from the reduced panels of biomarkers most strongly loaded onto the most important 10 biomarker components noted above (in Figure 1) decreased the proportion explained by biomarkers to 54% and 51% for each respective outcome in men, but made little difference for women (Figure 2B and Supplementary Table 5). These proportions were similar when adjusted for sociodemographic factors and health behaviors (Supplementary Table 5).

**Figure 3.**
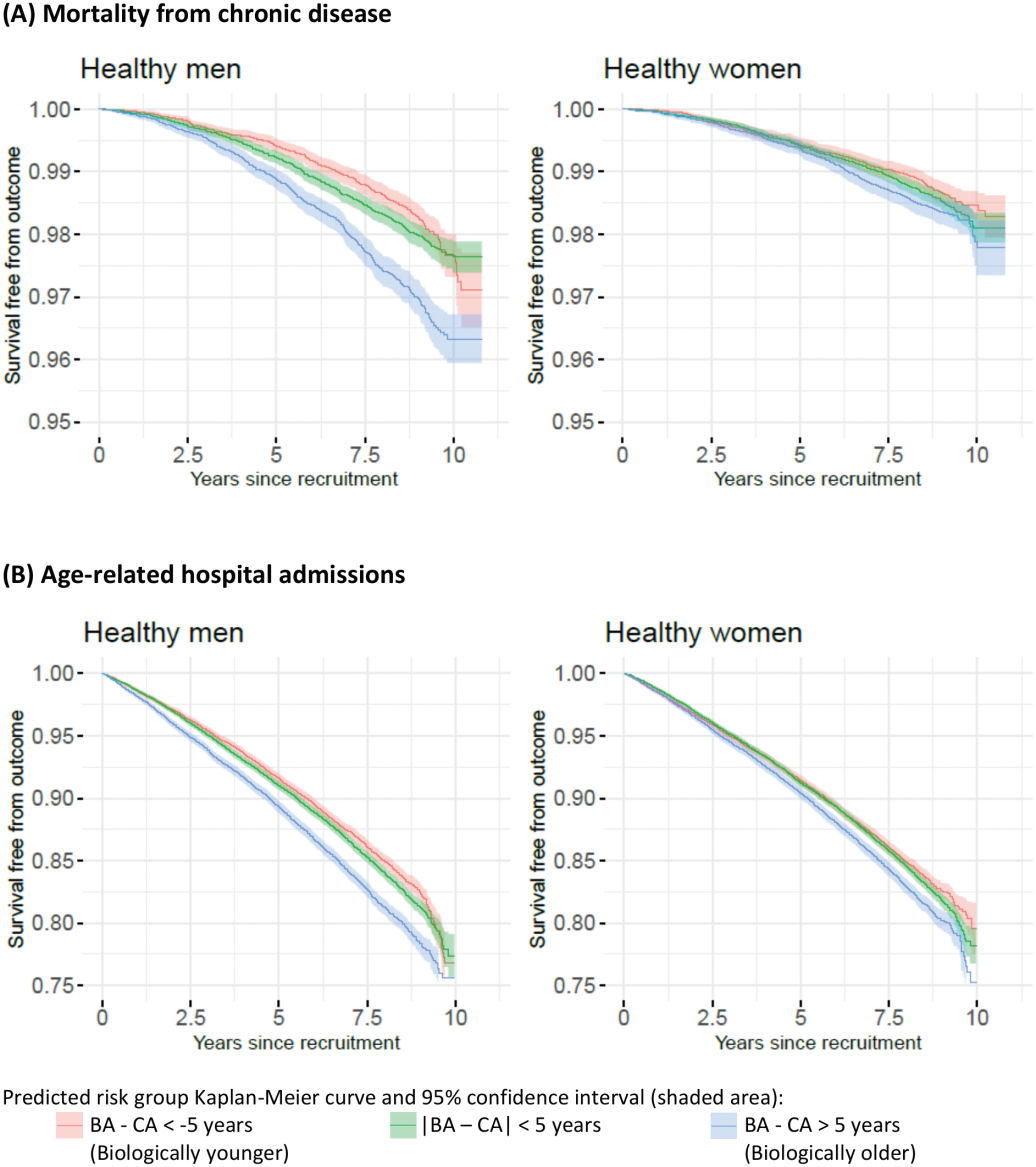
Outcome-free survival of healthy men and healthy women for (A) mortality from chronic disease and (B) age-related hospital admissions, according to whether their biomarker age is younger, similar to or older than their chronological age. *Note*: BA = biomarker age; CA = chronological age.

The biomarker ages identified 17.1% of healthy participants with a biomarker age ≥5 years younger and 16.9% with a biomarker age ≥5 years older than their chronological age (“biologically younger” vs “biologically older” participants). On aggregate, the mortality and hospital admission rates were highest in individuals who were biologically older and lowest in those who were biologically younger than their chronological age (Figure 3). At 8 years after the baseline date (approximate median follow-up), 1%/15% of biologically younger participants had died from chronic disease/had an age-related hospital admission, respectively, compared to 2%/18% of biologically older participants (based on the survival estimates in Figure 3). Log-rank tests of these survival differences had *p* values <1 × 10^−15^.

### Predictive Power of Biomarker Ages

In the healthy population, adding the biomarker age to a prediction model with chronological age increased the C-indices for mortality/hospital admission only slightly (0.008/0.003 in men, 0.002/0.001 in women; Table 2A). C-indices for the prediction of hospital admissions were greater for the biomarker ages than for the benchmark mortality score (difference in C-indices: 0.111/0.068 in men/women; Table 2A). Sociodemographic factors and health behaviors, which are adversely associated with health outcomes, were also associated with higher biomarker ages (living in an area classified within the most vs least deprived IMD quintile was associated with an extra 3.6/2.3 years of biomarker age for men/women after adjusting for chronological age alone), but adjustment for these factors did not substantially attenuate the differences in C-indices associated with adding the biomarker age into the prediction models with chronological age for men and women (Table 2B).

**Table 2.** Harrell’s C-Indices (with standard errors) for Each Health Outcome in the Healthy UK Biobank Subpopulation, Biomarker Age Versus Chronological Age, and Biomarker Age Versus Mortality Score

(A) Unadjusted Analysis		
Outcome and Age Predictor	Healthy Men	Healthy Women
Mortality from chronic disease		
CA alone	0.712 (0.008)	0.667 (0.009)
BA alone	0.689 (0.008)	0.635 (0.009)
BA and CA	0.720 (0.008)	0.669 (0.009)
*Improvement of BA and CA over CA*	*0.008*	*0.002*
Age-related hospital admissions		
CA alone	0.636 (0.003)	0.606 (0.003)
BA alone	0.615 (0.003)	0.586 (0.003)
BA and CA	0.639 (0.003)	0.608 (0.003)
*Improvement of BA and CA over CA*	*0.003*	*0.001*
Mortality score	0.504 (0.003)	0.518 (0.003)
*Improvement of BA over mortality score*	*0.111*	*0.068*
(B) Adjusted for Sociodemographic Factors and Health Behaviors		
Outcome and Age Predictor	Healthy Men	Healthy Women
Mortality from chronic disease		
CA alone	0.724 (0.008)	0.688 (0.009)
BA alone	0.702 (0.008)	0.660 (0.009)
BA and CA	0.731 (0.008)	0.690 (0.009)
*Improvement of BA and CA over CA*	*0.007*	*0.002*
Age-related hospital admissions		
CA alone	0.660 (0.003)	0.633 (0.003)
BA alone	0.640 (0.003)	0.614 (0.003)
BA and CA	0.662 (0.003)	0.634 (0.003)
*Improvement of BA and CA over CA*	*0.002*	*0.001*
Mortality score	0.574 (0.003)	0.571 (0.003)
*Improvement of BA over mortality score*	*0.066*	*0.043*

*Notes*: BA = biomarker age; CA = chronological age. Analyses in (B) were adjusted for Index of Multiple Deprivation 2010 quintile, smoking status, alcohol consumption, and assessment center. Figures in italics are differences in C-indices.

### Results for the Whole UK Biobank Population

In analyses run on the whole population, the importance of biomarker principal components in the biomarker ages was similar to that in healthy participants (Supplementary Figure 5 and Figure 1). The standard deviations of the differences between biomarker ages and chronological ages (9.7 years in men, 8.8 years in women) were slightly higher than those for the healthy subpopulation (8.7 years in men, 7.7 years in women). The biomarker ages explained greater proportions of the overall biomarker age and chronological age effect on on mortality and hospital admissions in the whole population (91%/84% for mortality/hospital admission, respectively, averaged across sexes; Supplementary Figure 6 vs Figure 2). Adding biomarker ages to the prediction models with chronological age statistically significantly improved model fit (*p* < 1 × 10^−10^ for both health outcomes and both sexes) in unadjusted analyses. Correspondingly, adding the biomarker ages to a prediction model with chronological age increased the C-indices for mortality/hospital admission (0.056/0.014 in men, 0.026/0.011 in women) more substantially than in the healthy population (Supplementary Table 6 vs Table 2).

## Discussion

This study found that the biomarker ages consisting of markers of impaired function in a range of organs accounted for >65% and >84% of the apparent effect of age on mortality and hospital admissions in the healthy and whole populations, respectively.

### Key Biomarker Determinants of Biomarker Ages and Their Relationships With Chronological Age

Lung, kidney, cognitive and liver function, IGF-1, hand grip strength, and blood pressure were key contributors to the biomarker ages for both sexes, while sex hormone-binding globulin and muscle mass in men, and cardiovascular function and HbA1c in women were also important.

These top-ranking biomarkers in this UK population generally matched those in a Singaporean study (8) and slight differences by sex were seen in both populations. However, these lung and renal function biomarkers were not investigated in the study comparing Canadian, South Korean, and Eastern European biological ages, which instead found that the top-ranking blood-based biomarkers varied by population and sex (14). Studies of aging biomarkers also found that lung and renal biomarkers were top-ranking determinants of functional decline (15) and variation in age-related traits (16). The present study provides additional detail on the relative importance on aging of biomarkers within body system groups, such as finding cystatin C to be more important than other renal biomarkers (creatinine and creatinine-based estimated glomerular filtration rate) (17), as previous studies each assessed only one of these biomarkers (8,14–16).

Several key biomarkers in this study (blood pressure, blood lipids, height, and lung function) have each been shown to be associated with a range of age-related diseases observationally, and in some cases causally in randomized trials and Mendelian randomization studies (Supplementary Table 7). Associations of other key biomarkers such as cystatin C and hand grip strength have been less extensively researched, but found to be associated with mortality or cardiovascular outcomes (17–21). Blood pressure (10th most important for healthy men and 8th for healthy women; Figure 1) is well-established as a modifiable and causal risk factor of cardiovascular disease (22).

### Prediction in Healthy Versus Unhealthier Individuals

In unhealthy individuals, their disease status and hospital admissions will already provide information of their risk (23), whereas, in apparently healthier individuals knowledge of a biomarker age is potentially more useful for identifying unrecognized health risks; furthermore, knowledge of risk of nonfatal outcomes should provide a longer window for intervention and prevention than knowledge of mortality risk. The biomarker ages were substantially better than the benchmark mortality score in predicting age-related hospital admissions (Table 2), and in the whole UK Biobank, the improvement in predictive power for mortality of the biomarker ages over chronological age (Table 2) and the effect sizes of the biomarker ages were comparable to the improvements reported by previous studies of biological ages in U.S., Canadian, and Singaporean populations (2,7,8,24). The biomarker ages had only slightly greater variation in the whole versus the healthy population, but the predictive value of the biomarker ages was considerably greater when including unhealthier individuals (Supplementary Table 6; Table 2). Hence, this could reflect a diagnostic element of these biomarkers that is stronger in the less healthy individuals. Therefore, when comparing biological ages across different studies it is important to take into account the health and age profile of populations.

Comparison of an individual’s biomarker age with their (unmodifiable) chronological age could provide a valuable means of communicating modifiable health risks, alongside their detailed biomarker profile (25). A biomarker age could also augment a national prevention program promoting clinical biomarker screening in a middle-aged population (26), after causal factors underlying its constituents have been established. The most important biomarkers in the biomarker ages were measured via blood biochemistry measurements, spirometry, and body size measurements, which can be administered routinely in clinical settings. For women, it may be suitable to measure just 12 key biomarkers (7 blood-based and 5 physical measurements; Figure 2B) across 7 body systems to assess biomarker aging, as relatively little explanatory and predictive value was compromised. A Healthy Aging Index constructed from a similar but smaller panel of biomarkers (blood pressure, lung function, creatinine, fasting glucose, and cognitive test biomarkers) was moderately predictive of mortality in a U.S. population (27). Despite the successful use of clinical risk prediction tools such as “heart age” and “lung age” in clinical care (28), there is little evidence as yet of implementation of an overall biological age, and proposals to use it in drug development (11,14,29) and clinical care (2,5,29) may be longer-term uses.

### Strengths and Limitations of This Study

The estimation methods used assumed that biomarkers with the strongest linear relation to chronological age contribute most to a biomarker age. Lung function biomarkers, systolic blood pressure, cystatin C, and reaction time had the strongest linear relationships with age (Supplementary Figure 2), and therefore contributed substantially to variation in the biomarker ages. However, a limitation of this approach is that not all biomarkers strongly associated with age may be reflective of clinical disease risk and, conversely, any risk factors for aging diseases that do not themselves have a strong relationship with age may have been underrepresented by the KDM. For example, body mass index has been causally linked to 30 diseases (including many age-related diseases) (30) but the general adiposity component was only 28th most important for men and 26th for women (Supplementary Table 4). Likewise, lipid-related cardiovascular biomarkers, LDL-C and apolipoprotein B are causally linked to atherosclerotic cardiovascular disease in men and women (31), but were only important in the biomarker age for women (Figure 1).

This approach was based on the cross-sectional associations of biomarkers with age, available for a large cohort, because repeat biomarker measurements (at one other time) were only available for a small subset of participants (10). However, in a study with multiple longitudinal measurements, the Pace of Aging, estimated from longitudinal changes in biomarkers over time, was shown to correlate with biological age estimated later (32).

The compositions of the derived biomarker ages were limited by the range of biomarkers available, and unlike the cohorts examined by studies of promising aging biomarkers (1,33,34), the UK Biobank is not specifically a gerontological resource. Cohort effects in this population are difficult to disentangle, and may influence trends in body size. Hence, height (1 of the top 15 most important biomarkers; Figure 1) may be acting as a proxy for cohort effects. Biomarker trends with age in the UK Biobank were not all completely linear (Supplementary Figure 2), but a previous study has shown that incorporating nonlinearity and nonmonotonicity (in limited functional forms) only slightly improved the accuracy of estimated biological age components, and was computationally complex (35). Moreover, biological ages estimated using KDM were found to be more predictive of mortality and frailty than biological ages estimated using machine learning methods in an older Singaporean population (8), and the only clinical biomarker-based age (14) identified by a review of deep learning biological aging scores (36) did not explore the improvement in predictive power from using a nonlinear estimation method. The epidemiological reliability of the present analyses was increased by focusing on a healthy subpopulation, using biomarker principal components and adherence to clinical risk prediction reporting guidelines (13) (Supplementary Table 8).

## Conclusions

Biomarker ages in men and women consisting of clinical biomarkers reflecting functionality of a range of organs accounted for a substantial proportion of the effects of age on disease and hospital admissions in the UK Biobank. They have the potential to be used and evaluated as a broader-based approach to risk identification and prevention than individual biomarkers. Of the most important biomarkers contributing to the derived biomarker ages, cardiometabolic biomarkers have well-studied causal associations with mortality and cardiovascular disease, but further research is needed to identify modifiable causal factors underlying all constituents of biological ages, for a range of age-related diseases.

## Supplementary Material

glab069_suppl_Supplementary_Materials

## Data Availability

The underlying data are open access through application to the UK Biobank, and materials and methods will be made freely available through the UK Biobank as part of this project.
