# Supplementary material for: A Biomarker-based Biological Age in UK Biobank: Composition and Prediction of Mortality and Hospital Admissions
Source: J Gerontol A Biol Sci Med Sci. 2021 Mar 6;76(7):1295–302. doi: 10.1093/gerona/glab069 (PMC8202154; doi:10.1093/gerona/glab069)
Supplement: glab069_suppl_Supplementary_Materials [file glab069_suppl_supplementary_materials.docx]

**Appendices – A biomarker-based biological age in UK Biobank: biomarker composition and prediction of mortality and hospital admissions**

(Short title: Biomarker age composition and outcome prediction)

Authors: Mei Sum Chan, Matthew Arnold, Alison Offer, Imen Hammami, Marion Mafham, Jane Armitage, Rafael Perera**,** Sarah Parish

Table of Contents

[Appendix 1. UK Biobank and study population 2](#_Toc55817777)

[UK Biobank 2](#_Toc55817778)

[Study population 2](#_Toc55817779)

[Identification of a healthy subpopulation 2](#_Toc55817780)

[Appendix 2. Exposure and outcome preparation 3](#_Toc55817781)

[Biomarker data cleaning 3](#_Toc55817782)

[Phenotyping health outcomes 3](#_Toc55817783)

[Appendix 3. Statistical analyses 5](#_Toc55817784)

[A. Analysis of biomarker characteristics 5](#_Toc55817785)

[B. Estimation of biomarker ages and a benchmark mortality score 5](#_Toc55817786)

[C. Biomarker importance in biomarker ages 6](#_Toc55817787)

[D. Prediction of adverse health outcomes 6](#_Toc55817788)

[Tables and figures 7](#_Toc55817789)

[eTable 1: List of the 72 UK Biobank biomarkers selected for analysis, with percentage of missing data for each biomarker in the whole population 7](#_Toc55817790)

[eTable 2: Constituent ICD-10 codes for the age-related hospital admissions definition, ranked by hazard ratio of baseline age in the UK Biobank 9](#_Toc55817791)

[eTable 3: Model coefficients for biomarker ages, in the healthy subpopulation, by sex 11](#_Toc55817792)

[eTable 4: Importance of each of the 51 biomarker principal components in the biomarker ages for healthy men and women 12](#_Toc55817793)

[eTable 5: Contributions of biomarker age and chronological age as a percentage of their total contribution to the explanation of each health outcome, in (A) the main analysis using the full biomarker panel and (B) when using the reduced biomarker panel 13](#_Toc55817794)

[eTable 6: Harrell's C-indices (with standard errors) for each health outcome in the whole UK Biobank population, biomarker age vs chronological age and biomarker age vs mortality score 14](#_Toc55817795)

[eTable 7: Examples of associations in published studies between the top 10 biomarker principal components of biomarker age identified in the present study and adverse health outcomes 15](#_Toc55817796)

[eTable 8: Transparent Reporting of a multivariable prediction model for Individual Prognosis Or Diagnosis (TRIPOD) checklist for this study 16](#_Toc55817797)

[eFigure 1: Flowchart of selection of study population, before population stratification 18](#_Toc55817798)

[eFigure 2: Biomarker-age trends for the 72 candidate biomarkers, healthy men vs healthy women 19](#_Toc55817799)

[eFigure 3: Characterisation of the first 51 biomarker principal components 22](#_Toc55817800)

[eFigure 4: Means and standard deviations of biomarker ages by 2.5-year chronological age groups, for healthy men and healthy women 31](#_Toc55817801)

[eFigure 5: Importance of the top 15 biomarker principal components in the biomarker ages for men and women in the whole UK Biobank population 32](#_Toc55817802)

[eFigure 6: Relative contribution of biomarker ages and chronological age in explaining each health outcome, for men and women in the whole UK Biobank population 33](#_Toc55817803)

[eFigure 7: Outcome-free survival of men and women in the whole UK Biobank population for (A) mortality from chronic disease and (B) age-related hospital admissions, according to whether their biomarker age is younger, similar to or older than their chronological age 34](#_Toc55817804)

[Supplementary References 35](#_Toc55817805)

# Appendix 1. UK Biobank and study population

## UK Biobank

The UK Biobank is a large and richly phenotyped prospective study with over 500,000 participants aged 40-70 years when recruited in 2006–2010 (1). The resource has collected and continues to collect extensive phenotypic and genotypic detail about its participants, including data from questionnaires, physical measures, sample assays, and longitudinal follow-up for a wide range of health-related outcomes (1). Details on the recruitment, biomarker measurement and data linkage procedures are available from the UK Biobank website (2). UK Biobank is an open access data resource for bona fide researchers who wish to use it to conduct health-related research for the benefit of the public, and access procedures are also detailed on the UK Biobank website (2).

## Study population

This study included all UK Biobank participants in the April 2019 data extract. Information on sociodemographic characteristics, self-reported health behaviours, health ratings and medication were collected from touchscreen questionnaires. Linkage to Hospital Episode Statistics (HES) provided prior and prospective information on secondary care outcomes. Linkage to the Office for National Statistics (ONS) death registry provided date and cause of death. Biomarkers used in this study were measured via physical measurement devices, blood assays and urine assays. Sex, age (rounded down to month) and date of assessment were available for all participants. Index of Multiple Deprivation (IMD) 2010 score was grouped into quintiles within the UK Biobank population in each country. Of the 502,536 participants in the UK Biobank, participants were excluded if they had no date of assessment or did not attend the verbal interview, had neither blood count nor blood serum biochemistry measurements, were younger than 40 or older than 70 years at baseline, or had no IMD score. After these exclusions, there were 480,019 participants in the study population (eFigure 1).

Participants attended baseline assessment in 2006-2010 and a subset of ≈20,000 participants attended a repeat assessment in 2012-2013 (1). Participants were followed up for a median of 8.7 years to the death record censoring date of 31 January 2018 for English and Welsh participants or 30 November 2016 for Scottish participants. HES records were available for a median follow up of 8.0 years, until 31 March 2017, 31 October 2016 or 29 February 2016 for English, Scottish, or Welsh participants respectively.

## Identification of a healthy subpopulation

A healthy subpopulation of the UK Biobank was identified from a composite measure of prior health: no chronic disease medications, good health, steady/brisk walk speed, never/ex-smoker (self-reported in touchscreen questionnaire); 0-2 secondary care episodes prior to recruitment, and no age-related prior disease or hip/wrist fracture (recorded in HES), based on the following approach. The 1366 self-reported medication names were classified as chronic disease-related using a data-driven approach based on the British National Formulary mapping and clinician review (3). Diseases in scope as ‘age-related prior disease’ were those chronic diseases defined using secondary care ICD-10 codes (in main or secondary diagnostic positions within the records) phenotyped by a previous study of chronic disease incidence in English adults (4) that were age-related based on the criteria: 1. commonly classified as a chronic rather than acute disease, 2. clear increasing trend in incidence with chronological age, and 3. not defined solely by candidate biomarker levels. The diseases included by these criteria were: cardiac arrhythmia, chronic kidney disease, diabetes mellitus, heart failure, ischaemic heart disease, peripheral arterial disease, arthritis, rheumatoid arthritis, osteoporosis, gout, dementia, stroke/transient ischaemic attack, chronic obstructive pulmonary disease, connective tissue disease, liver disease and malignant cancers.

In addition, prior hip and wrist fractures (ICD-10 codes S72 and S62 respectively) were also included as age-related prior disease.

# Appendix 2. Exposure and outcome preparation

## Biomarker data cleaning

As at April 2019, 110 physical and biochemical biomarkers were available in UK Biobank. Biomarkers were excluded from the panel for this study if they were measured in <70% of the whole population, if they were not measured on a continuous scale, or if they measured the same biological trait (e.g. standardly-measured but not impedance device-measured weight was selected), leaving 74 biomarkers. A further 2 biomarkers, oestradiol and nucleic red blood cell count, were excluded due to poor reproducibility (intra-individual Pearson correlation coefficient adjusted for baseline age of ≤0.1 in the repeat assessment subset of 19,335 participants). The remaining 72 biomarkers were categorised by body system (eTable 1), based partially on the biomarker categorisation used by a review of biological age studies (5).

Biomarker measurements were cleaned as follows:

1. The UK Biobank-defined ‘best measure’ readings for the lung function biomarkers, forced expiratory volume in 1 second (FEV1) and forced vital capacity (FVC) (2), were supplemented by raw readings flagged as ‘acceptable’ or deemed to be reproducible in the repeat assessment, to reduce their missingess from 29% to 9% (as has been advocated (6)). FEV1, FVC, and hand grip strength were each divided by standing height to reduce dependence on body size (7).
2. Urinary and blood serum biomarkers, flagged at assay as below or above the assay reportable range (8) were replaced with the respective limit of the range.
3. All biomarkers, values were standardised by subtracting their overall mean and dividing by their standard deviation. Body size biomarkers exhibited sex differences, therefore they were standardised separately within each sex.
4. Standardised values outside ±4 were treated as outliers, set to missing, and subsequently imputed in step 4.
5. Imputation of standardised biomarkers was carried out by replacing all missing values with the overall medians within 5-year baseline age, similar to a procedure based on imputing overall means (9), preferred to assigning a non-central biomarker value through multiple imputation (10) and complete case analysis (11-14). Relative to chronological age, multiple imputation would skew individuals’ biomarker ages in the direction indicated by the available biomarkers, and the degree of statistical inference applied to missing biomarkers and the resulting uncertainty in biomarker age estimation would not be represented in their biomarker age.
6. Sensitivity tests on steps 3–4 where biomarker values were transformed using rank-based inverse normal transform and the threshold for outliers was modified to standardised values outside ±10 had a negligible effect on the correlations of each biomarker with chronological age (change in Pearson correlation coefficients <0.0005).

## Phenotyping health outcomes

Two adverse health outcomes were investigated in this study and were phenotyped from death registry and HES records, based on code lists and procedures published by previous studies (9, 11, 12, 15).

1. Mortality due to chronic disease:

Mortality is the most objective and most accurately recorded outcome available in UK Biobank. Accidental deaths (11, 12) and non-chronic disease deaths (9) were excluded, following previous studies’ procedures. The exclusions were specified by ICD-10 Chapter: certain infectious and parasitic diseases (A00-B99), pregnancy, childbirth and the puerperium (O00-O99), congenital malformations, deformations and chromosomal abnormalities (Q00-Q99), injury, poisoning and certain other consequences of external causes (S00-T98) and external causes of morbidity and mortality (V01-Y98).

1. First admission to hospital with an age-related diagnosis:

Hospital diagnoses relating to symptoms of biological frailty were identified through a candidate list of ICD-10 codes used in a frailty risk score for secondary care records (15). As not all components typically included in frailty assessment instruments are age-related or recorded in secondary care diagnoses (18), for the avoidance of confusion the term ‘hospital admissions’ was used instead of ‘frailty’.

The age-related hospital admisssion outcome in this study was constructed from the candidate ICD-10 codelist in the hospital frailty risk score study (15):

1. Incident cases in the UK Biobank at April 2017 were identified for each of 75 candidate frailty subtypes (standalone 3-digit ICD-10 codes in main or secondary diagnoses with >500 incident cases, otherwise grouped ICD-10 codes within the same ICD-10 block (eTable 2)).
2. Hazard ratios per 10 years of baseline chronological age were then estimated for each candidate subtype as the outcome, with Cox models adjusted for smoking status, alcohol intake frequency and Townsend deprivation quintile.
3. Subtypes were included in the definition if this hazard ratio exceeded the threshold of 1.2.

The number of events for each outcome was summarised by subpopulation and sex in Table 1.

# Appendix 3. Statistical analyses

## A. Analysis of biomarker characteristics

Biomarker-age trends were assessed for linearity and for homogeneity between sexes and across prior health subpopulations. To estimate biomarker-age trends, linear regression was used to obtain least-square means and standard errors of standardised biomarker values by 2.5-year chronological age groups, separately by sex, adjusted for Index of Multiple Deprivation 2010 quintile, smoking status, alcohol consumption band and assessment centre. Trends for each biomarker were visually assessed for linearity across age groups (Figure 1 and eFigure 2), as subsequent statistical methods assume linearity of biomarker-biomarker or biomarker-age relationships.

Many previous studies used biomarker-age correlations to pre-select biomarkers for inclusion into biological ages (11-13, 19, 20). Pre-selection was not carried out in this study, to avoid selecting biomarkers that are potentially highly correlated with each other (due to their high correlation with chronological age), and to allow the methods for estimating biomarker age to conduct their own selection process.

## B. Estimation of biomarker ages and a benchmark mortality score

Previous studies that compared several estimation methods applied to clinical biomarkers (11, 21, 22) reported that biomarker ages estimated using the Klemera Doubal method (KDM) (23) appeared to have the highest predictive power for health outcomes, followed by multiple linear regression (MLR), then Principal Component Analysis (PCA). A review of biomarker age estimation methods (5) that compared statistical properties and limitations of these three methods listed more limitations in the MLR and PCA methods than in the KDM.

The PCA method was integrated with the KDM (21), to reduce the effect of biomarker multicollinearity in the KDM. PCA was first used to summarise the biomarkers into linearly independent biomarker principal components (24). To aid clinical interpretation, varimax rotation (21) was applied after PCA. The rotated principal components were individually characterised based on the relative contributions of their constituent biomarkers, measured via rotated factor loadings (eFigure 3). The rotated factor loadings and the eigenvalues of the principal components were similar when run on the healthy subpopulation and the whole population, thus only the results for the whole population were used, for consistency in interpretation.

Biomarker ages were then estimated using the KDM separately by sex, due to differences in biomarker-age trends by sex (eFigure 2). Additionally, mortality from chronic disease, prior and incident age-related hospital admissions were different between sexes (Table 1). The KDM assumes that its constituent biomarkers are uncorrelated and is based on two principles: 1. The biomarker age summarises the differences between individuals’ actual biomarker levels $x_{j}$, where *j = 1, …, m* for *m* candidate biomarkers, and characteristic biomarker levels for their chronological age; and 2. biomarkers with stronger linear relationships to chronological age contribute more to the biomarker age (23). Biomarker age was estimated by linearly regressing each included biomarker principal component $x_{j}$ against chronological age, then taking the weighted sum of all the regression results, with the form:

$$Biological age\propto\sum_{j=1}^{m} \left( \frac{k_{j}}{s_{j}^{2}} \right)\left( x_{j}-q_{j} \right)$$

where $q_{j}$ = intercept, $k_{j}$ = coefficient and $s_{j}$=standard error from the j^th^ biomarker-chronological age regression

Some studies found a second version of KDM that included chronological age as a biomarker controversial (14, 21). To assess biomarker ages both in isolation and jointly with chronological age, only the version of KDM without chronological age as a biomarker was used.

Previous studies had derived a mortality-based score (25) and biological age (26) using Cox proportional hazards models with variable selection. As a sensitivity test, a benchmark mortality score was derived using the same biomarker panel for consistency with this study, using a Bonferroni-corrected stepwise Cox model similar to the previously published score (25). The predictive power of the mortality score and the biomarker ages for age-related hospital admissions, in the absence of other risk factors, were compared using Harrell’s C-indices from unadjusted Cox models stratified by sex (Appendix 3D).

## C. Biomarker importance in biomarker ages

The relative importance of each biomarker was calculated as the proportion of variance in the biomarker ages explained by each constituent biomarker in the presence of the other constituent biomarkers (R^2^). This was derived using the Fabbris/Genizi/Johnson method (27) implemented in the R package ‘relaimpo’ (28), as recommended by a review of relative importance estimation methods when there are large numbers of variables (29).

## D. Prediction of adverse health outcomes

For each of the 2 outcomes defined in Appendix 2, Cox proportional hazards models were run on the same populations as those used in the biomarker age estimation. For models that predicted age-related hospital admissions, participants with prior events were excluded. These models were stratified by sex, and the age combinations used in each model were: 1. chronological age, 2. biomarker age, and 3. both chronological and biomarker age. Unadjusted models and models adjusted for IMD quintile, smoking status, alcohol consumption and assessment centre were run. The improvement in model fit by adding biomarker age to the prediction model with chronological age was assessed using a likelihood ratio χ^2^ test with 1 degree of freedom.

Predictive power was assessed using Harrell’s C-index, a measure of statistical discrimination for survival models that is equivalent to area under the receiver operating characteristic curve. The C-index and its standard errors were calculated using Kendall’s tau (30).

The proportion of variation in chronological age explained by each biomarker age was estimated in terms of R^2^ from univariate linear regressions. The proportion of the combined age effect on either mortality or hospital admission risk that was explained by biomarker age was estimated by comparing the log partial likelihoods of pairs of nested models (an extension of likelihood ratio tests):

$$\frac{\left( l_{BA+CA}-l_{CA} \right)}{\left( l_{BA+CA}-l_{base} \right)}$$

where *l_m_*: log-likelihood of model *m*, *base*: model without chronological or biomarker age, *BA*: model with biomarker age only and *BA+CA*: model with both biomarker and chronological age

Similar ratios were taken to estimate the proportion explained by chronological age alone, and by the overlap of biological and chronological age. The log-likelihood proportions above are equivalent to comparisons of the Nagelkerke pseudo-R^2^ (31) of the same pairs of models, which are approximations of R^2^ for Cox models.

All statistical analyses were run in R version 3.3.3.

# Tables and figures

### eTable 1: List of the 72 UK Biobank biomarkers selected for analysis, with percentage of missing data for each biomarker in the whole population

| **No.** | **Body system group** | **Biomarker description** | **% missing** |
| --- | --- | --- | --- |
| 1 | **Cardiovascular:** | Diastolic blood pressure | 0.1 |
| 2 |  | Systolic blood pressure | 0.1 |
| 3 |  | Pulse rate | 0.1 |
| 4 |  | Apolipoprotein A | 12.9 |
| 5 |  | Apolipoprotein B | 5.0 |
| 6 |  | Lipoprotein (a) | 7.6 |
| 7 |  | High density lipoprotein cholesterol | 12.7 |
| 8 |  | Low density lipoprotein cholesterol | 4.9 |
| 9 |  | Triglycerides | 4.7 |
| 10 | **Clotting:** | Mean platelet volume | 2.9 |
| 11 |  | Platelet count | 2.9 |
| 12 |  | Platelet crit | 2.9 |
| 13 |  | Platelet distribution width | 2.9 |
| 14 | **Endocrine, metabolic** | Log C-Reactive Protein | 4.8 |
| 15 | **and immune:** | Blood glucose | 12.8 |
| 16 |  | HbA1c | 5.3 |
| 17 |  | Insulin-like growth factor 1 | 5.2 |
| 18 |  | Sex hormone-binding globulin | 13.4 |
| 19 |  | Testosterone | 5.6 |
| 20 | **Liver:** | Albumin | 12.7 |
| 21 |  | Alanine aminotransferase | 4.7 |
| 22 |  | Aspartate aminotransferase | 5.1 |
| 23 |  | Direct bilirubin | 7.5 |
| 24 |  | Total bilirubin | 5.1 |
| 25 |  | Gamma Glutamyltransferase | 4.7 |
| 26 | **Musculoskeletal:** | Heel bone density | 1.8 |
| 27 |  | Body mass index* | 0.4 |
| 28 |  | Sitting height* | 0.3 |
| 29 |  | Standing height* | 0.3 |
| 30 |  | Hip circumference* | 0.2 |
| 31 |  | Waist circumference* | 0.2 |
| 32 |  | Waist-hip ratio* | 0.2 |
| 33 |  | Weight* | 0.3 |
| 34 |  | Body fat-free mass* | 1.8 |
| 35 |  | Body fat mass* | 1.9 |
| 36 |  | Body fat percentage* | 1.8 |
| 37 |  | Metabolic rate* | 1.8 |
| 38 |  | Hand grip strength/height* | 0.4 |
| 39 |  | Alkaline Phosphatase | 4.7 |
| 40 |  | Calcium | 12.7 |
| 41 |  | Rheumatoid factor | 4.7 |
| 42 |  | Vitamin D | 8.5 |
| 43 | **Nervous:** | Reaction time test | 1.1 |
| 44 |  | Pairs matching test | 3.5 |
| 45 | **Red blood cells:** | Haemoglobin concentration | 2.9 |
| 46 |  | HLS reticulocyte count | 4.6 |
| 47 |  | Immature reticulocyte fraction | 4.6 |
| 48 |  | Mean corpuscular volume | 2.9 |
| 49 |  | Mean reticulocyte volume | 4.6 |
| 50 |  | Mean spherical cell volume | 4.6 |
| 51 |  | Total red blood cell count | 2.9 |
| 52 |  | Red blood cell distribution width | 2.9 |
| 53 |  | Reticulocyte count | 4.6 |
| 54 |  | Mean corpuscular haemoglobin concentration | 2.9 |

| **No.** | **Body system group** | **Biomarker description** | **% missing** |
| --- | --- | --- | --- |
| 55 | **Renal:** | Urinary microalbumin | 2.9 |
| 56 |  | Urinary sodium | 2.9 |
| 57 |  | Urinary creatinine | 2.7 |
| 58 |  | Urinary potassium | 2.9 |
| 59 |  | Urea | 4.8 |
| 60 |  | Creatinine | 4.7 |
| 61 |  | Cystatin C | 4.7 |
| 62 |  | Phosphate | 12.8 |
| 63 |  | Total protein | 12.8 |
| 64 |  | Urate | 4.8 |
| 65 | **Respiratory:** | Forced expiratory volume in 1s/height* | 8.9 |
| 66 |  | Forced vital capacity/height* | 8.9 |
| 67 | **White blood cells:** | Eosinophil count | 3.1 |
| 68 |  | Lymphocyte count | 3.1 |
| 69 |  | Monocyte count | 3.1 |
| 70 |  | Neutrophil count | 3.1 |
| 71 |  | Basophil count | 3.1 |
| 72 |  | Total white blood cell count | 2.9 |

Note: * Values were standardised separately for men and women, due to large sex differences.

All biochemical biomarkers were measured via blood assays unless labelled as ‘urinary’.

### eTable 2: Constituent ICD-10 codes for the age-related hospital admissions definition, ranked by hazard ratio of baseline age in the UK Biobank

| **No** | **ICD10 group** | **ICD-10 codes** | **Incident cases in UK Biobank** | **Hazard ratio for 10 years of age** |
| --- | --- | --- | --- | --- |
| 1 | Dementia | F00 F01 F03 G30 | 214 | 5.70 |
| 2 | Parkinsons | G20 | 576 | 3.16 |
| 3 | Chronic renal failure | N18 | 873 | 3.14 |
| 4 | Osteoporosis without pathological fracture | M81 | 1454 | 2.66 |
| 5 | Other disorders of fluid, electrolyte and acid-base balance | E87 | 554 | 2.29 |
| 6 | Retention of urine | R33 | 1274 | 2.28 |
| 7 | Transient cerebral ischaemic attacks and related syndromes | G45 | 508 | 2.27 |
| 8 | Delirium | F05 | 54 | 2.27 |
| 9 | Polyarthrosis | M15 | 913 | 2.19 |
| 10 | Respiratory disease not infection | J69 J96 | 412 | 2.17 |
| 11 | Cerebrovascular | I67 I69 | 794 | 2.11 |
| 12 | Osteoporosis | M80 | 546 | 2.07 |
| 13 | Cerebral Infarction | I63 | 574 | 2.07 |
| 14 | Other hearing loss | H91 | 864 | 2.00 |
| 15 | Other abnormal findings of blood chemistry | R79 | 1816 | 1.90 |
| 16 | Renal failure | N17 N19 | 956 | 1.90 |
| 17 | Neurodegenerative disease | G31 | 114 | 1.90 |
| 18 | Problems related to social environment | Z60 | 839 | 1.86 |
| 19 | Skin ulcer | L89 L97 | 308 | 1.83 |
| 20 | Kidney urinary disorders | N28 | 876 | 1.82 |
| 21 | Other arthrosis | M19 | 4403 | 1.80 |
| 22 | Spinal stenosis (secondary code only) | M48 | 1038 | 1.73 |
| 23 | Digestive disease | K26 | 1567 | 1.65 |
| 24 | Pneumonia, organism unspecified | J18 | 1256 | 1.60 |
| 25 | Blindness or low vision | H54 | 381 | 1.60 |
| 26 | Dorsophathy | M41 | 379 | 1.59 |
| 27 | Fall on same level from slipping, tripping and stumbling | W01 | 1597 | 1.59 |
| 28 | Unspecified fall | W19 | 926 | 1.57 |
| 29 | Hypotension | I95 | 717 | 1.51 |
| 30 | Syncope and collapse | R55 | 1541 | 1.49 |
| 31 | Metabolic disorder | E83 E86 | 1135 | 1.47 |
| 32 | Cognition emotion behaviour symptoms | R40 R41 R44 R45 R47 | 1577 | 1.47 |
| 33 | Symptoms and signs concerning food and fluid intake | R63 | 1405 | 1.45 |
| **No** | **ICD10 group** | **ICD-10 codes** | **Incident cases in UK Biobank** | **Hazard ratio for 10 years of age** |
| 34 | Other external | Y84 Y95 Z22 Z50 Z73 Z74 Z75 Z93 Z99 | 3954 | 1.44 |
| 35 | Hemiplegia | G81 | 381 | 1.43 |
| 36 | Fall | W06 W18 | 850 | 1.39 |
| 37 | Urinary system symptoms | R32 | 918 | 1.37 |
| 38 | Unspecified acute lower respiratory infection | J22 | 998 | 1.36 |
| 39 | Nervous and musculoskeletal symptoms | R26 R29 | 1000 | 1.35 |
| 40 | Other bacterial agents as the cause of diseases classified to other chapters (secondary code) | B96 | 1051 | 1.33 |
| 41 | Fall on and from stairs and steps | W10 | 639 | 1.32 |
| 42 | Unspecified haematuria | R31 | 3447 | 1.31 |
| 43 | Abnormalities of heart beat | R00 | 1939 | 1.30 |
| 44 | Personal history of other diseases and conditions | Z87 | 6111 | 1.26 |
| 45 | Skin infection | L08 | 627 | 1.26 |
| 46 | Infection | A04 A41 B95 | 3444 | 1.25 |
| 47 | Other anaemias | D64 | 2237 | 1.25 |
| 48 | Dysphagia | R13 | 1554 | 1.25 |
| 49 | Pancreatic disorder | E16 | 230 | 1.23 |
| 50 | Abnormal results of function studies | R94 | 755 | 1.22 |
| 51 | Other functional intestinal disorders | K59 | 1955 | 1.22 |
| 52 | Gangrene | R02 | 137 | 1.22 |

Note: This codelist excludes cancer or any form of neoplasms.

P-values of hazard ratios for 10 years of age for each ICD-10 group were significant at the 10^-3^ level.

### eTable 3: Model coefficients for biomarker ages, in the healthy subpopulation, by sex

| **Biomarker principal component number and description** | | **Healthy men** | | | **Healthy women** | | |
| --- | --- | --- | --- | --- | --- | --- | --- |
|  |  | **q_j_** | **k_j_** | **s_j_** | **q_j_** | **k_j_** | **s_j_** |
| PC1 | General adiposity | 55.224 | 0.004 | 11.865 | 56.673 | -1.035 | 10.628 |
| PC2 | Total haemoglobin volume | 56.276 | 1.035 | 11.814 | 58.763 | -3.455 | 10.344 |
| PC3 | Height | 56.007 | 3.638 | 10.626 | 56.429 | 3.049 | 10.038 |
| PC4 | Albumin | 56.235 | 5.479 | 10.676 | 55.896 | -0.074 | 10.896 |
| PC5 | Neutrophil count | 55.806 | -1.896 | 11.633 | 56.036 | -0.543 | 10.875 |
| PC6 | Immature red blood cell volume | 55.353 | -2.078 | 11.565 | 55.880 | 0.151 | 10.894 |
| PC7 | LDL and ApoB | 55.047 | -0.746 | 11.824 | 55.293 | -4.247 | 9.166 |
| PC8 | Reticulocyte count | 55.226 | 0.055 | 11.865 | 56.530 | -1.526 | 10.739 |
| PC9 | Urinary potassium and creatinine | 55.248 | -0.101 | 11.864 | 55.950 | 0.251 | 10.893 |
| PC10 | Blood pressure | 54.700 | -2.940 | 11.374 | 56.959 | -3.768 | 9.829 |
| PC11 | HDL and ApoA | 55.715 | 0.980 | 11.811 | 55.797 | 0.126 | 10.895 |
| PC12 | Aminotransferases | 55.303 | 0.325 | 11.860 | 57.384 | -4.235 | 10.206 |
| PC13 | Bilirubin | 55.759 | 1.016 | 11.774 | 55.432 | 1.558 | 10.719 |
| PC14 | Platelet count | 55.067 | -0.343 | 11.858 | 55.798 | 0.319 | 10.888 |
| PC15 | Red blood cell haemoglobin concentration | 55.442 | -1.244 | 11.798 | 55.889 | -0.048 | 10.896 |
| PC16 | Testosterone | 54.896 | -0.385 | 11.862 | 56.129 | -0.345 | 10.896 |
| PC17 | Lung function/height | 57.442 | -5.247 | 9.508 | 57.285 | -4.819 | 8.697 |
| PC18 | Blood glucose | 55.467 | 2.362 | 11.694 | 56.254 | 3.519 | 10.508 |
| PC19 | Platelet cell volume | 55.216 | -0.181 | 11.864 | 55.929 | -0.722 | 10.869 |
| PC20 | LP(a) | 55.233 | 0.081 | 11.865 | 55.886 | 0.906 | 10.858 |
| PC21 | Pairs matching test | 55.541 | -3.107 | 11.464 | 56.001 | -2.285 | 10.670 |
| PC22 | Rheumatoid factor | 55.234 | 0.597 | 11.851 | 55.898 | 0.455 | 10.887 |
| PC23 | Bone density | 55.485 | -0.830 | 11.836 | 55.451 | -2.792 | 10.587 |
| PC24 | Vitamin D | 55.180 | 0.897 | 11.832 | 55.882 | 0.242 | 10.893 |
| PC25 | IGF-1 | 56.478 | 5.561 | 10.692 | 56.011 | 3.770 | 10.242 |
| PC26 | Urinary microalbumin | 55.491 | 3.197 | 11.636 | 56.257 | 2.829 | 10.767 |
| PC27 | Basophil count | 55.356 | 1.306 | 11.809 | 55.893 | -0.153 | 10.895 |
| PC28 | Central adiposity | 56.393 | -4.109 | 11.274 | 56.605 | -3.698 | 10.338 |
| PC29 | Eosinophil count | 55.214 | 0.838 | 11.836 | 56.041 | 0.829 | 10.869 |
| PC30 | Alkaline phosphatase | 55.614 | -2.022 | 11.738 | 56.426 | -5.813 | 9.215 |
| PC31 | Pulse rate | 55.576 | -1.408 | 11.783 | 55.926 | -1.888 | 10.763 |
| PC32 | Red blood cell width | 55.694 | -3.190 | 11.552 | 55.904 | -0.203 | 10.894 |
| PC33 | Reaction time test | 56.417 | -5.222 | 10.788 | 55.963 | -4.210 | 10.109 |
| PC34 | Sex hormone-binding globulin | 58.143 | 7.049 | 11.096 | 56.932 | -2.355 | 10.600 |
| PC35 | Hand grip strength/height | 56.156 | 5.354 | 10.716 | 56.783 | 5.022 | 9.845 |
| PC36 | Phosphate | 54.881 | 1.169 | 11.806 | 55.469 | -2.109 | 10.724 |
| PC37 | Lymphocyte count | 55.340 | -0.547 | 11.855 | 55.902 | -2.055 | 10.722 |
| PC38 | Triglycerides | 55.226 | 0.008 | 11.865 | 57.879 | 6.395 | 9.838 |
| PC39 | Urinary sodium | 55.393 | 0.634 | 11.847 | 55.754 | 0.477 | 10.888 |
| PC40 | Monocyte count | 54.981 | 2.936 | 11.544 | 56.276 | 1.301 | 10.841 |
| PC41 | Gamma glutamyltransferase | 55.145 | 0.680 | 11.847 | 57.554 | 4.670 | 10.325 |
| PC42 | Urea | 54.943 | 2.177 | 11.690 | 56.967 | 4.759 | 9.985 |
| PC43 | HbA1c | 56.395 | 4.928 | 11.232 | 57.306 | 6.937 | 9.638 |
| PC44 | Platelet distribution width | 55.217 | 0.071 | 11.865 | 55.964 | 0.583 | 10.882 |
| PC45 | Log C-reactive protein | 56.097 | 4.298 | 11.264 | 56.620 | 4.315 | 10.172 |
| PC46 | Reticulocyte fraction | 55.424 | 1.087 | 11.821 | 56.028 | 0.913 | 10.862 |
| PC47 | Cystatin C | 55.163 | -6.091 | 10.883 | 58.861 | -7.437 | 9.137 |
| PC48 | Muscle mass | 55.919 | -6.416 | 10.722 | 56.018 | -4.356 | 10.485 |
| PC49 | Calcium | 55.023 | -3.212 | 11.488 | 55.788 | 3.217 | 10.429 |
| PC50 | Total protein | 55.330 | 2.042 | 11.695 | 55.903 | -1.683 | 10.770 |
| PC51 | Urate | 54.829 | 0.870 | 11.845 | 59.192 | 6.360 | 9.978 |

Note: q_j_ = intercept, k_j_ = coefficient and s_j_ = standard error from the j^th^ biomarker-chronological age regression.

###

### eTable 4: Importance of each of the 51 biomarker principal components in the biomarker ages for healthy men and women

| **Healthy men** | | |  | | | |  | | **Healthy women** | | | | |  | | |
| --- | --- | --- | --- | --- | --- | --- | --- | --- | --- | --- | --- | --- | --- | --- | --- | --- |
|  | |  | | |  |  | | | | | |  | | | |  |
| **Rank** | **Biomarker principal component** | | | **Proportion of total R^2^ (%)** | | | |  | | **Rank** | **Biomarker principal component** | | **Proportion of total R^2^ (%)** | |  |  |
| 1 | Lung function/height | | | 12.4 | | | |  | | 1 | Lung function/height | | 10.3 | |  |  |
| 2 | Reaction time test | | | 6.9 | | | |  | | 2 | Cystatin C | | 8.0 | |  |  |
| 3 | IGF-1 | | | 6.7 | | | |  | | 3 | LDL and ApoB | | 7.0 | |  |  |
| 4 | Cystatin C | | | 6.7 | | | |  | | 4 | Alkaline phosphatase | | 6.6 | |  |  |
| 5 | Hand grip strength/height | | | 6.4 | | | |  | | 5 | HbA1c | | 5.9 | |  |  |
| 6 | Albumin | | | 6.3 | | | |  | | 6 | Hand grip strength/height | | 5.6 | |  |  |
| 7 | Sex hormone-binding globulin | | | 6.0 | | | |  | | 7 | Urea | | 4.9 | |  |  |
| 8 | Muscle mass | | | 5.9 | | | |  | | 8 | Blood pressure | | 4.9 | |  |  |
| 9 | Height | | | 5.6 | | | |  | | 9 | Reaction time test | | 4.6 | |  |  |
| 10 | Blood pressure | | | 3.5 | | | |  | | 10 | IGF-1 | | 4.0 | |  |  |
| 11 | HbA1c | | | 3.5 | | | |  | | 11 | Height | | 3.8 | |  |  |
| 12 | Central adiposity | | | 2.9 | | | |  | | 12 | Triglycerides | | 3.7 | |  |  |
| 13 | Pairs matching test | | | 2.6 | | | |  | | 13 | Urate | | 3.2 | |  |  |
| 14 | Log C-reactive protein | | | 2.5 | | | |  | | 14 | Aminotransferases | | 2.8 | |  |  |
| 15 | Calcium | | | 2.1 | | | |  | | 15 | Log C-reactive protein | | 2.2 | |  |  |
| 16 | Immature red blood cell volume | | | 2.0 | | | |  | | 16 | Bone density | | 2.2 | |  |  |
| 17 | Red blood cell width | | | 1.8 | | | |  | | 17 | Total haemoglobin volume | | 2.1 | |  |  |
| 18 | Total protein | | | 1.7 | | | |  | | 18 | Gamma glutamyltransferase | | 1.9 | |  |  |
| 19 | Monocyte count | | | 1.5 | | | |  | | 19 | Blood glucose | | 1.9 | |  |  |
| 20 | Urea | | | 1.5 | | | |  | | 20 | Central adiposity | | 1.8 | |  |  |
| 21 | Urinary microalbumin | | | 1.4 | | | |  | | 21 | Muscle mass | | 1.8 | |  |  |
| 22 | Blood glucose | | | 1.2 | | | |  | | 22 | Calcium | | 1.7 | |  |  |
| 23 | Neutrophil count | | | 1.1 | | | |  | | 23 | Pairs matching test | | 1.3 | |  |  |
| 24 | HDL and ApoA | | | 0.8 | | | |  | | 24 | Phosphate | | 1.0 | |  |  |
| 25 | Vitamin D | | | 0.6 | | | |  | | 25 | Sex hormone-binding globulin | | 0.8 | |  |  |
| 26 | Alkaline phosphatase | | | 0.6 | | | |  | | 26 | General adiposity | | 0.8 | |  |  |
| 27 | Total haemoglobin volume | | | 0.6 | | | |  | | 27 | Urinary microalbumin | | 0.7 | |  |  |
| 28 | General adiposity | | | 0.5 | | | |  | | 28 | Bilirubin | | 0.7 | |  |  |
| 29 | Urinary sodium | | | 0.5 | | | |  | | 29 | Lymphocyte count | | 0.5 | |  |  |
| 30 | LDL and ApoB | | | 0.4 | | | |  | | 30 | Pulse rate | | 0.4 | |  |  |
| 31 | Phosphate | | | 0.4 | | | |  | | 31 | Reticulocyte count | | 0.4 | |  |  |
| 32 | Pulse rate | | | 0.4 | | | |  | | 32 | Total protein | | 0.3 | |  |  |
| 33 | Bilirubin | | | 0.4 | | | |  | | 33 | HDL and ApoA | | 0.3 | |  |  |
| 34 | Red blood cell haemoglobin concentration | | | 0.3 | | | |  | | 34 | Urinary sodium | | 0.3 | |  |  |
| 35 | Testosterone | | | 0.3 | | | |  | | 35 | Albumin | | 0.2 | |  |  |
| 36 | Platelet count | | | 0.3 | | | |  | | 36 | LP(a) | | 0.2 | |  |  |
| 37 | Basophil count | | | 0.3 | | | |  | | 37 | Testosterone | | 0.2 | |  |  |
| 38 | Urate | | | 0.2 | | | |  | | 38 | Vitamin D | | 0.2 | |  |  |
| 39 | Reticulocyte count | | | 0.2 | | | |  | | 39 | Monocyte count | | 0.1 | |  |  |
| 40 | Bone density | | | 0.2 | | | |  | | 40 | Platelet cell volume | | 0.1 | |  |  |
| 41 | Aminotransferases | | | 0.2 | | | |  | | 41 | Platelet distribution width | | 0.1 | |  |  |
| 42 | Triglycerides | | | 0.2 | | | |  | | 42 | Urinary potassium and creatinine | | 0.1 | |  |  |
| 43 | Reticulocyte fraction | | | 0.2 | | | |  | | 43 | Neutrophil count | | 0.1 | |  |  |
| 44 | Gamma glutamyltransferase | | | 0.1 | | | |  | | 44 | Reticulocyte fraction | | 0.1 | |  |  |
| 45 | Rheumatoid factor | | | 0.1 | | | |  | | 45 | Platelet count | | 0.1 | |  |  |
| 46 | Eosinophil count | | | 0.1 | | | |  | | 46 | Eosinophil count | | 0.1 | |  |  |
| 47 | Lymphocyte count | | | 0.1 | | | |  | | 47 | Red blood cell width | | 0.1 | |  |  |
| 48 | Urinary potassium and creatinine | | | 0.1 | | | |  | | 48 | Rheumatoid factor | | 0.1 | |  |  |
| 49 | Platelet distribution width | | | 0.0 | | | |  | | 49 | Immature red blood cell volume | | 0.1 | |  |  |
| 50 | Platelet cell volume | | | 0.0 | | | |  | | 50 | Basophil count | | 0.0 | |  |  |
| 51 | LP(a) | | | 0.0 | | | |  | | 51 | Red blood cell haemoglobin concentration | | 0.0 | |  |  |

### eTable 5: Contributions of biomarker age and chronological age as a percentage of their total contribution to the explanation of each health outcome, in (A) the main analysis using the full biomarker panel and (B) when using the reduced biomarker panel

**(A) Full biomarker panel**

|  |  | **Death from chronic disease** | | | **Age-related hospital admissions** | | |
| --- | --- | --- | --- | --- | --- | --- | --- |
|  |  | **CA alone** | **CA and BA** | **BA alone** | **CA alone** | **CA and BA** | **BA alone** |
| **Unadjusted analysis** | **Healthy men** | 27.0 | 64.0 | 9.0 | 33.1 | 62.1 | 4.8 |
|  | **Healthy women** | 38.2 | 58.8 | 3.0 | 36.1 | 61.2 | 2.8 |
|  | **All men** | 4.5 | 50.5 | 45.0 | 17.9 | 63.2 | 18.9 |
|  | **All women** | 13.1 | 58.7 | 28.2 | 14.7 | 65.0 | 20.2 |
| **Adjusted analysis** | **Healthy men** | 28.3 | 63.5 | 8.2 | 34.7 | 61.4 | 4.0 |
|  | **Healthy women** | 39.0 | 58.3 | 2.7 | 39.6 | 58.7 | 1.7 |
|  | **All men** | 8.3 | 56.6 | 35.1 | 20.8 | 64.5 | 14.7 |
|  | **All women** | 18.9 | 60.8 | 20.4 | 18.8 | 66.1 | 15.1 |

**(B) Using the reduced biomarker panel**

|  |  | **Death from chronic disease** | | | **Age-related hospital admissions** | | |
| --- | --- | --- | --- | --- | --- | --- | --- |
|  |  | **CA alone** | **CA and BA** | **BA alone** | **CA alone** | **CA and BA** | **BA alone** |
| **Unadjusted analysis** | **Healthy men** | 46.2 | 50.1 | 3.8 | 49.5 | 48.2 | 2.4 |
|  | **Healthy women** | 35.3 | 60.1 | 4.7 | 37.7 | 59.5 | 2.8 |
|  | **All men** | 16.0 | 51.4 | 32.6 | 38.8 | 52.1 | 9.1 |
|  | **All women** | 14.9 | 59.3 | 25.8 | 17.3 | 64.5 | 18.2 |
| **Adjusted analysis** | **Healthy men** | 47.0 | 49.9 | 3.1 | 50.0 | 48.2 | 1.9 |
|  | **Healthy women** | 35.0 | 60.3 | 4.7 | 39.8 | 58.2 | 2.0 |
|  | **All men** | 23.0 | 55.2 | 21.9 | 42.5 | 51.7 | 5.8 |
|  | **All women** | 20.4 | 61.3 | 18.3 | 21.2 | 65.1 | 13.6 |

Note: CA: chronological age, BA: biomarker age. These proportions were estimated by comparing the log partial likelihoods of pairs of nested models (an extension of likelihood ratio tests; Appendix 3D). Models used in the adjusted analyses were adjusted for Index of Multiple Deprivation 2010 quintile, smoking status, alcohol consumption and assessment centre.

### eTable 6: Harrell's C-indices (with standard errors) for each health outcome in the whole UK Biobank population, biomarker age vs chronological age and biomarker age vs mortality score

**(A) Unadjusted analysis**

| **Outcome and age predictor** | **Men** | **Women** |
| --- | --- | --- |
| **Mortality from chronic disease** |  |  |
| CA alone | 0.686 (0.003) | 0.670 (0.003) |
| BA alone | 0.736 (0.003) | 0.683 (0.003) |
| BA and CA | 0.742 (0.003) | 0.696 (0.003) |
| *Improvement of BA and CA over CA* | *0.056* | *0.026* |
| **Age-related hospital admissions** |  |  |
| CA alone | 0.629 (0.003) | 0.603 (0.003) |
| BA alone | 0.63 (0.003) | 0.605 (0.003) |
| BA and CA | 0.643 (0.003) | 0.614 (0.003) |
| *Improvement of BA and CA over CA* | *0.014* | *0.011* |
| Mortality score | 0.501 (0.003) | 0.506 (0.003) |
| *Improvement of BA over mortality score* | *0.129* | *0.099* |

**(B) Adjusted for sociodemographic factors and health behaviours**

| **Outcome and age predictor** | **Men** | **Women** |
| --- | --- | --- |
| **Mortality from chronic disease** |  |  |
| CA alone | 0.725 (0.003) | 0.699 (0.003) |
| BA alone | 0.746 (0.003) | 0.697 (0.003) |
| BA and CA | 0.756 (0.003) | 0.713 (0.003) |
| *Improvement of BA and CA over CA* | *0.031* | *0.014* |
| **Age-related hospital admissions** |  |  |
| CA alone | 0.677 (0.001) | 0.656 (0.001) |
| BA alone | 0.673 (0.001) | 0.653 (0.001) |
| BA and CA | 0.685 (0.001) | 0.662 (0.001) |
| *Improvement of BA and CA over CA* | *0.008* | *0.006* |
| Mortality score | 0.605 (0.001) | 0.602 (0.001) |
| *Improvement of BA over mortality score* | *0.068* | *0.052* |

Note: CA: chronological age; BA: biomarker age. Analyses in (B) were adjusted for Index of Multiple Deprivation 2010 quintile, smoking status, alcohol consumption and assessment centre.

### eTable 7: Examples of associations in published studies between the top 10 biomarker principal components of biomarker age identified in the present study and adverse health outcomes

| **Key biomarker principal component in the present study** | **Evidence from meta-analyses of randomised trials** | **Evidence from Mendelian randomisation** | **Evidence from prospective studies** |
| --- | --- | --- | --- |
| Lung function/height | - | Respiratory and autoimmune diseases (32) | All-cause, circulatory disease, respiratory and cancer mortality (6) |
| Cystatin C | - | (Not significantly associated with cardiovascular disease (33)) | Cardiovascular disease, mortality (34, 35) and end-stage renal disease (35) |
| Reaction time test | - | - | Mortality (36) |
| IGF-1 | Fracture risk (37) | (Not significantly associated with Alzheimer’s disease (38)) | Mortality and heart failure (39) and cognitive function (40) |
| Hand grip strength/height | - | Cardiovascular disease and mortality (41) | Mortality, cardiovascular disease, respiratory disease, cancer (42) |
| Blood pressure | Mortality and cardiovascular disease (43) | Type 2 diabetes (44), Alzheimer’s disease (45) | Vascular mortality (associations weaken with age) (46) |
| Albumin (men only) | - | - | Coronary heart disease (47) |
| Sex hormone-binding globulin (men only) | - | Type 2 diabetes (48) | Type 2 diabetes (49) |
| Muscle mass (men only) | - | - | Cancer mortality (50), physical disability (51) |
| Height (men only) | - | Cardiovascular disease, hip fracture, intervertebral disc disorder, vasculitis, gastro-oesophageal reflux disease and cancer (52) | Mortality from cardiovascular disease, liver disease, COPD, stomach and oral cancers, mental disorders (53) |
| LDL-C and ApoB (women only) | Major vascular disease, vascular and all-cause mortality (54) | Coronary heart disease (55) | Cardiovascular disease (56) |
| Alkaline phosphatase (women only) | - | Type 2 diabetes (57) (Associations with IHD and T2D not robust after allowing for pleiotropy (58)) | Osteosarcoma (59) and cardiovascular disease (60) |
| HbA1c (women only) | (Not significantly associated with cardiovascular disease (61)) | Coronary artery disease (62) | Mortality, cardiovascular disease, cancer, diabetes (63) |
| Urea (women only) | - | - | Coronary heart disease (64) |

### eTable 8: Transparent Reporting of a multivariable prediction model for Individual Prognosis Or Diagnosis (TRIPOD) checklist for this study

| **Section/Topic** | **Item*** | | **Checklist Item** | **Page** |
| --- | --- | --- | --- | --- |
| **Title and abstract** | | | | |
| Title | 1 | D;V | Identify the study as developing and/or validating a multivariable prediction model, the target population, and the outcome to be predicted. | p1 |
| Abstract | 2 | D;V | Provide a summary of objectives, study design, setting, participants, sample size, predictors, outcome, statistical analysis, results, and conclusions. | p2 |
| **Introduction** | | | | |
| Background and objectives | 3a | D;V | Explain the medical context (including whether diagnostic or prognostic) and rationale for developing or validating the multivariable prediction model, including references to existing models. | Appendix p5-6 |
|  | 3b | D;V | Specify the objectives, including whether the study describes the development or validation of the model or both. | p4-7 |
| **Methods** | | | | |
| Source of data | 4a | D;V | Describe the study design or source of data (e.g., randomized trial, cohort, or registry data), separately for the development and validation data sets, if applicable. | Appendix p2 |
|  | 4b | D;V | Specify the key study dates, including start of accrual; end of accrual; and, if applicable, end of follow-up. | Appendix p2 |
| Participants | 5a | D;V | Specify key elements of the study setting (e.g., primary care, secondary care, general population) including number and location of centres. | Appendix p2-3 |
|  | 5b | D;V | Describe eligibility criteria for participants. | Appendix p2 |
|  | 5c | D;V | Give details of treatments received, if relevant. | NA |
| Outcome | 6a | D;V | Clearly define the outcome that is predicted by the prediction model, including how and when assessed. | Appendix p3 |
|  | 6b | D;V | Report any actions to blind assessment of the outcome to be predicted. | NA |
| Predictors | 7a | D;V | Clearly define all predictors used in developing or validating the multivariable prediction model, including how and when they were measured. | Appendix p3-5 |
|  | 7b | D;V | Report any actions to blind assessment of predictors for the outcome and other predictors. | NA |
| Sample size | 8 | D;V | Explain how the study size was arrived at. | Appendix p2 |
| Missing data | 9 | D;V | Describe how missing data were handled (e.g., complete-case analysis, single imputation, multiple imputation) with details of any imputation method. | Appendix p3 |
| Statistical analysis methods | 10a | D | Describe how predictors were handled in the analyses. | Appendix p4 |
|  | 10b | D | Specify type of model, all model-building procedures (including any predictor selection), and method for internal validation. | Appendix p5-6 |
|  | 10c | V | For validation, describe how the predictions were calculated. | Appendix p6 |
|  | 10d | D;V | Specify all measures used to assess model performance and, if relevant, to compare multiple models. | Appendix p5-6 |
|  | 10e | V | Describe any model updating (e.g., recalibration) arising from the validation, if done. | NA |
| Risk groups | 11 | D;V | Provide details on how risk groups were created, if done. | Appendix p5 |
| Development vs. validation | 12 | V | For validation, identify any differences from the development data in setting, eligibility criteria, outcome, and predictors. | NA |

| **Section/Topic** | **Item*** | **Checklist Item** | **Page** |
| --- | --- | --- | --- |

| **Results** | | | | |
| --- | --- | --- | --- | --- |
| Participants | 13a | D;V | Describe the flow of participants through the study, including the number of participants with and without the outcome and, if applicable, a summary of the follow-up time. A diagram may be helpful. | Appendix p2, eFigure 1 |
|  | 13b | D;V | Describe the characteristics of the participants (basic demographics, clinical features, available predictors), including the number of participants with missing data for predictors and outcome. | Appendix p2-3 |
|  | 13c | V | For validation, show a comparison with the development data of the distribution of important variables (demographics, predictors and outcome). | NA |
| Model development | 14a | D | Specify the number of participants and outcome events in each analysis. | Table 1 |
|  | 14b | D | If done, report the unadjusted association between each candidate predictor and outcome. | NA |
| Model specification | 15a | D | Present the full prediction model to allow predictions for individuals (i.e., all regression coefficients, and model intercept or baseline survival at a given time point). | eTable 3 |
|  | 15b | D | Explain how to use the prediction model. | Appendix p5-6 |
| Model performance | 16 | D;V | Report performance measures (with CIs) for the prediction model. | Table 2 |
| Model-updating | 17 | V | If done, report the results from any model updating (i.e., model specification, model performance). | NA |
| **Discussion** | | | | |
| Limitations | 18 | D;V | Discuss any limitations of the study (such as nonrepresentative sample, few events per predictor, missing data). | p13 |
| Interpretation | 19a | V | For validation, discuss the results with reference to performance in the development data, and any other validation data. | NA |
|  | 19b | D;V | Give an overall interpretation of the results, considering objectives, limitations, results from similar studies, and other relevant evidence. | p10-14 |
| Implications | 20 | D;V | Discuss the potential clinical use of the model and implications for future research. | p12-14 |
| **Other information** | | | | |
| Supplementary information | 21 | D;V | Provide information about the availability of supplementary resources, such as study protocol, Web calculator, and data sets. | Appendix p2 |
| Funding | 22 | D;V | Give the source of funding and the role of the funders for the present study. | Funding section |

Note: This study is a development and internal validation study under TRIPOD guidelines (65).

* Items relevant to model development are denoted by ‘D’, items relating to model validation are denoted by ‘V’.

### eFigure 1: Flowchart of selection of study population, before population stratification

UK Biobank participants in the main dataset

(n = 502,536)

Exclude participants with no date of assessment or who have not attended the verbal interview (n = 841)

Step 1 dataset

(n = 501,695)

Exclude participants who had neither blood count nor serum measurements (n = 9106)

Step 2 dataset

(n = 492,589)

Exclude participants younger than 40 or older than 70 years at baseline (n = 14)

Step 3 dataset

(n = 492,575)

Exclude participants with missing Index of Multiple Deprivation score (n = 12,556)

Cleaned UK Biobank dataset

(n = 480,019)

### eFigure 2: Biomarker-age trends for the 72 candidate biomarkers, healthy men vs healthy women


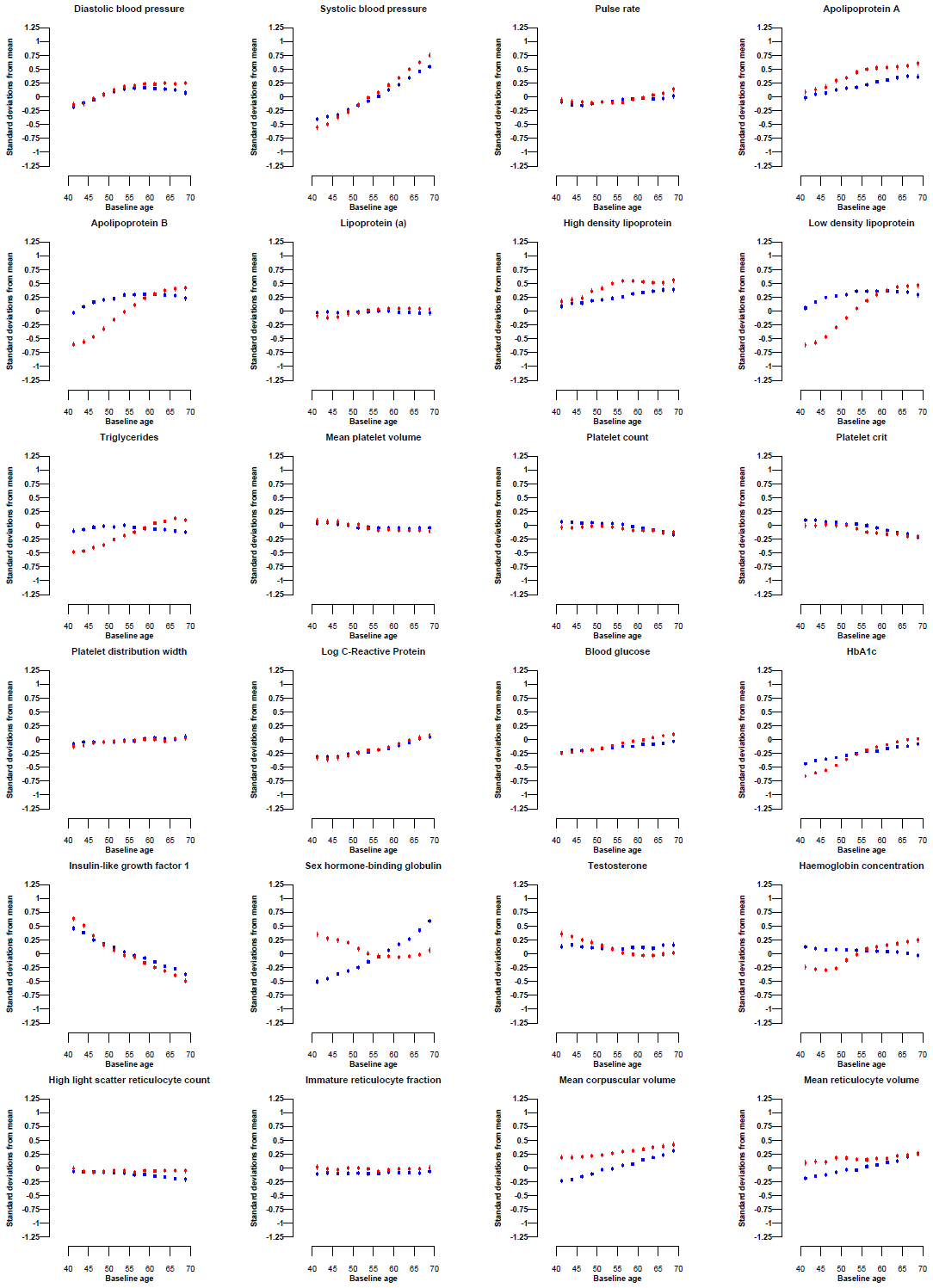


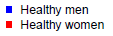


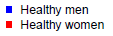

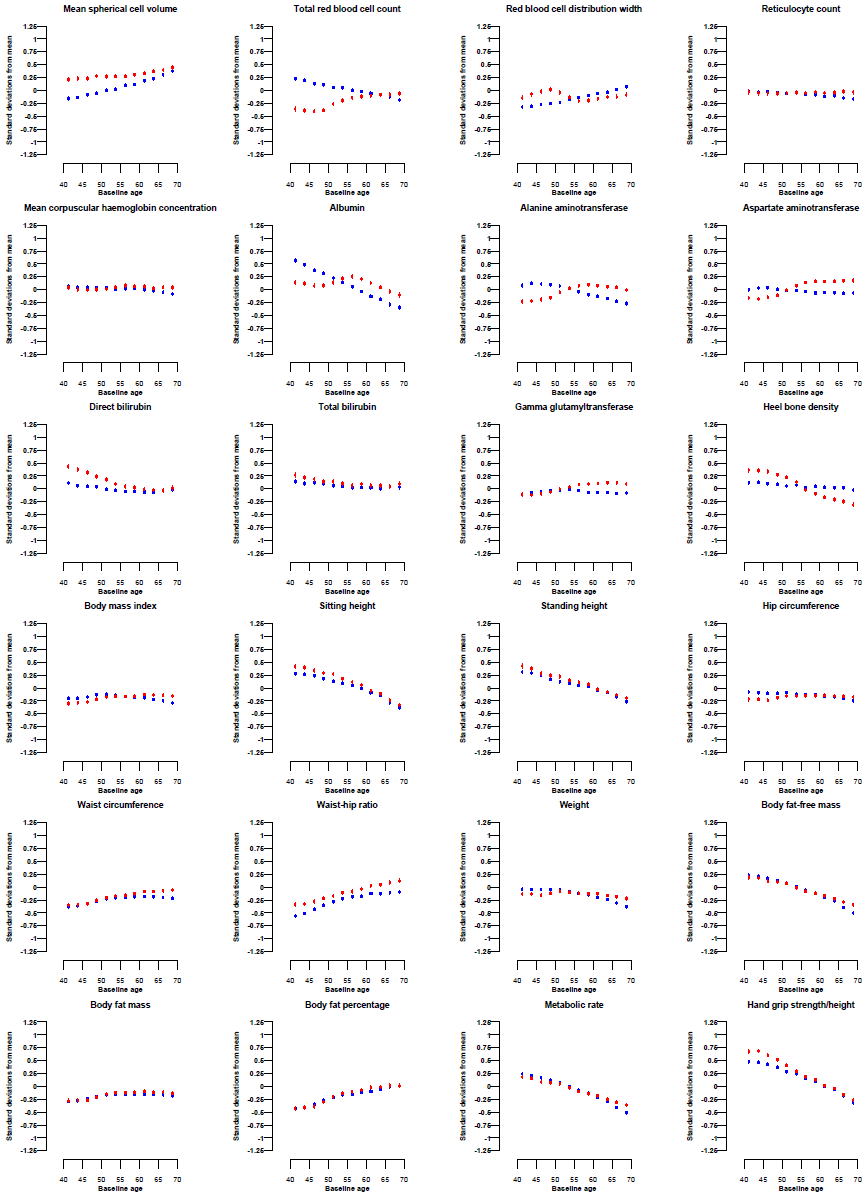


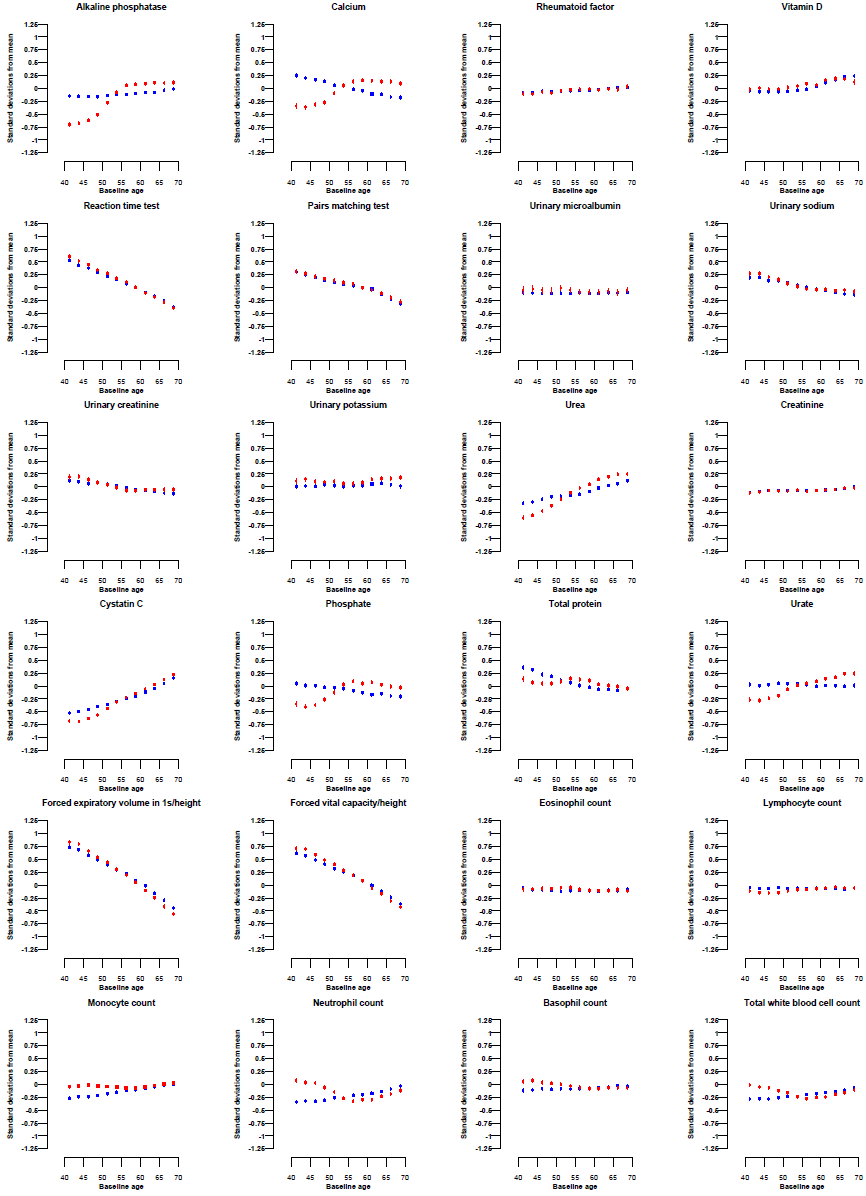


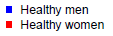


### eFigure 3: Characterisation of the first 51 biomarker principal components


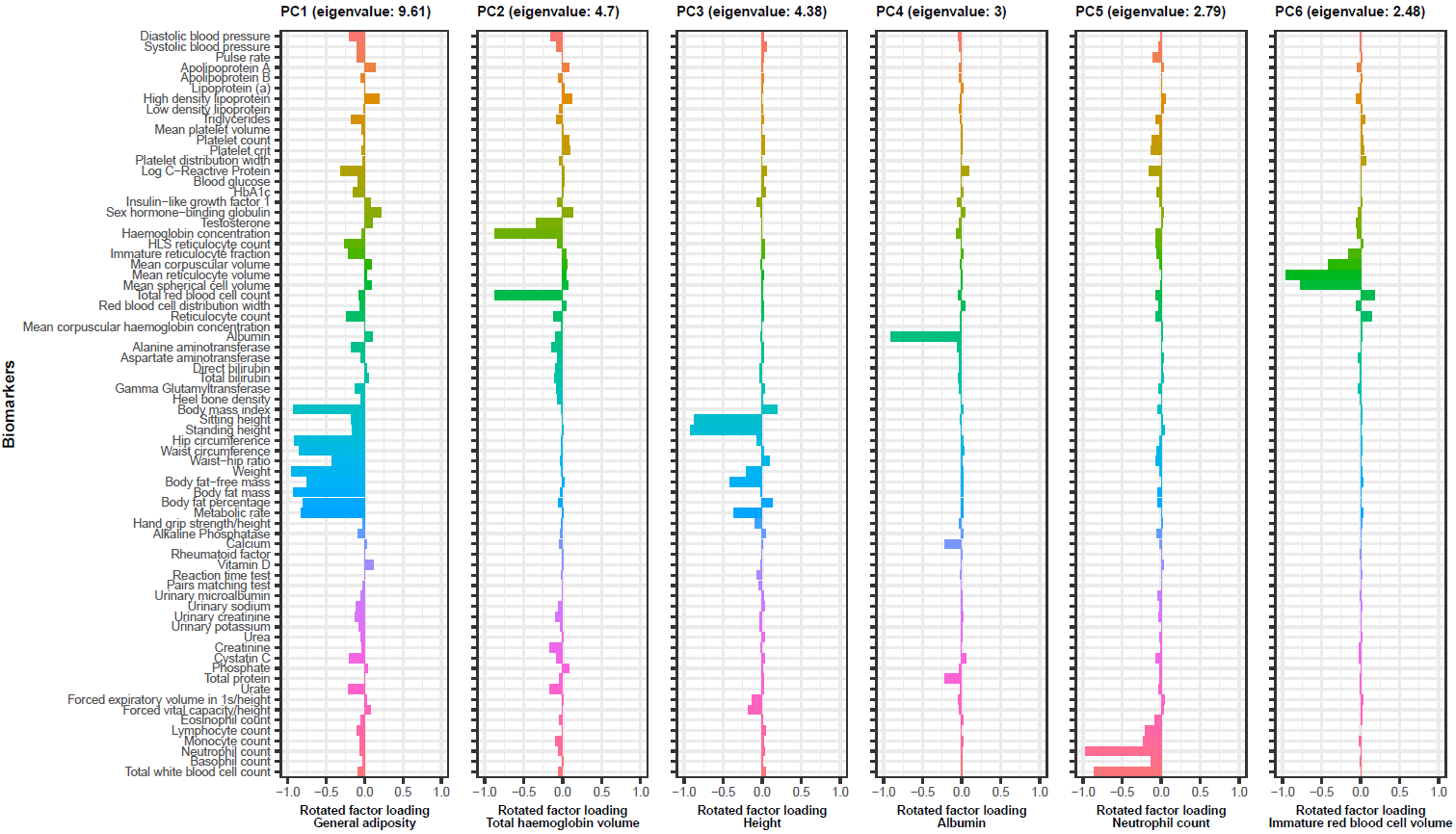

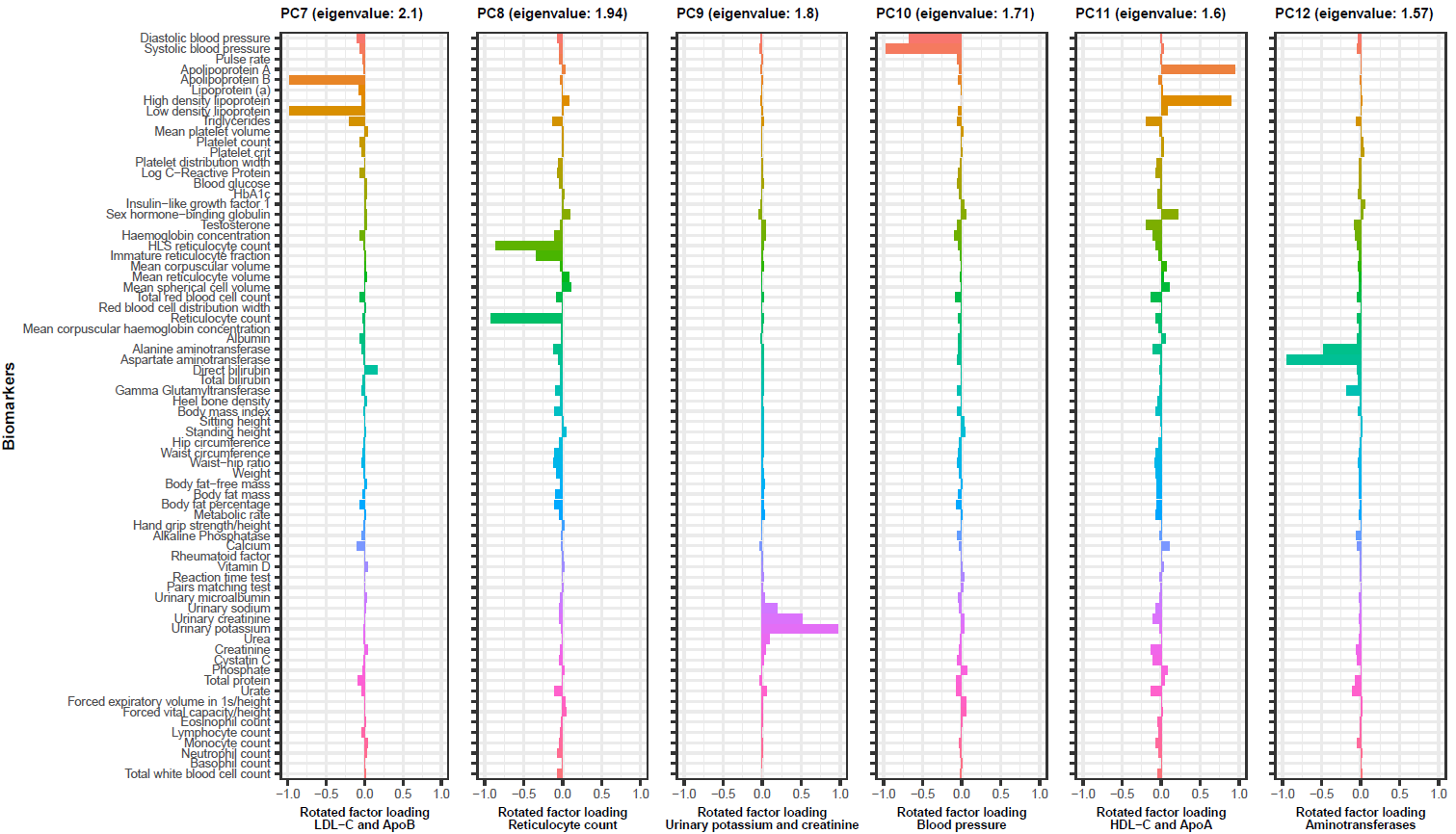

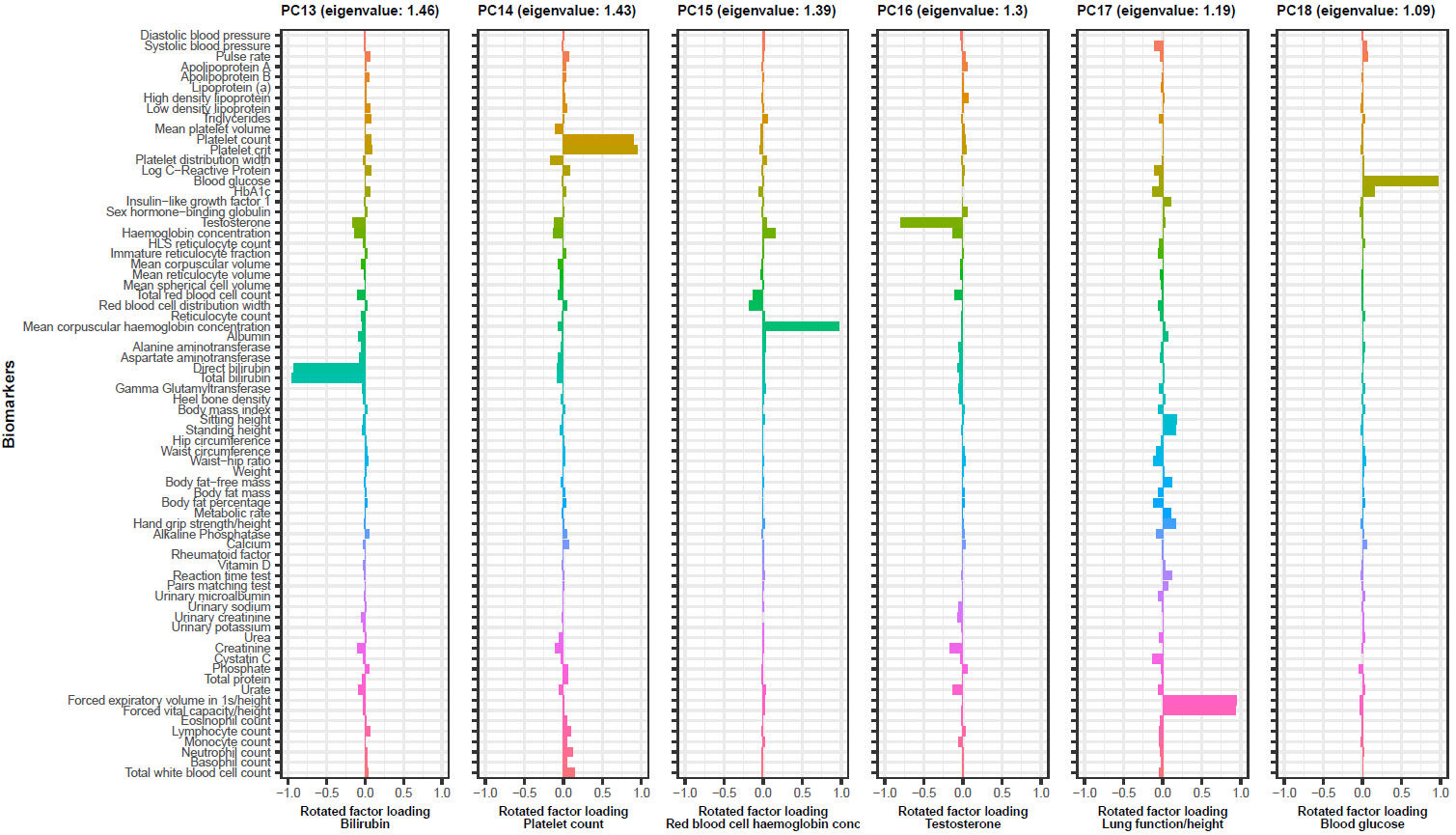

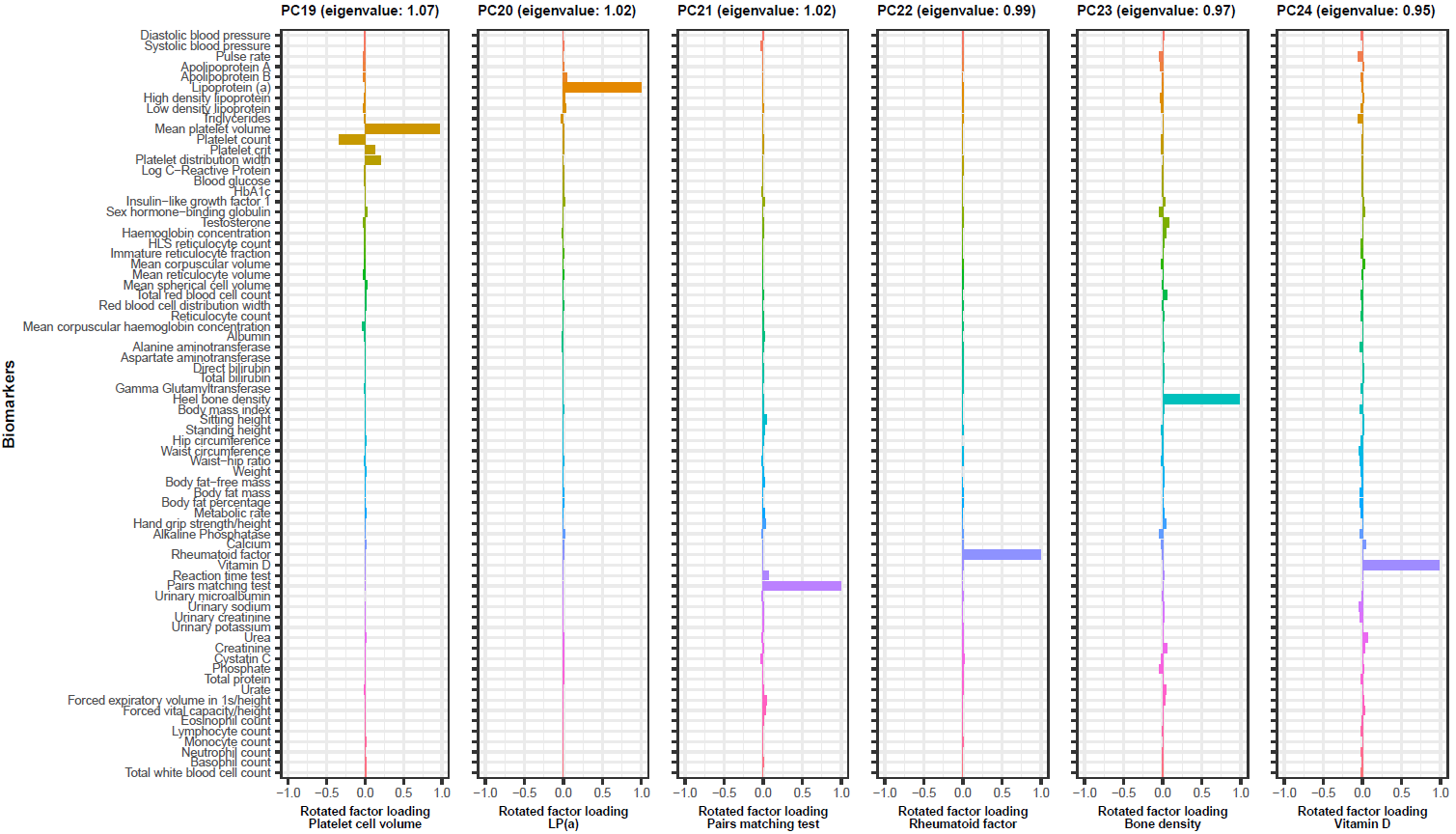

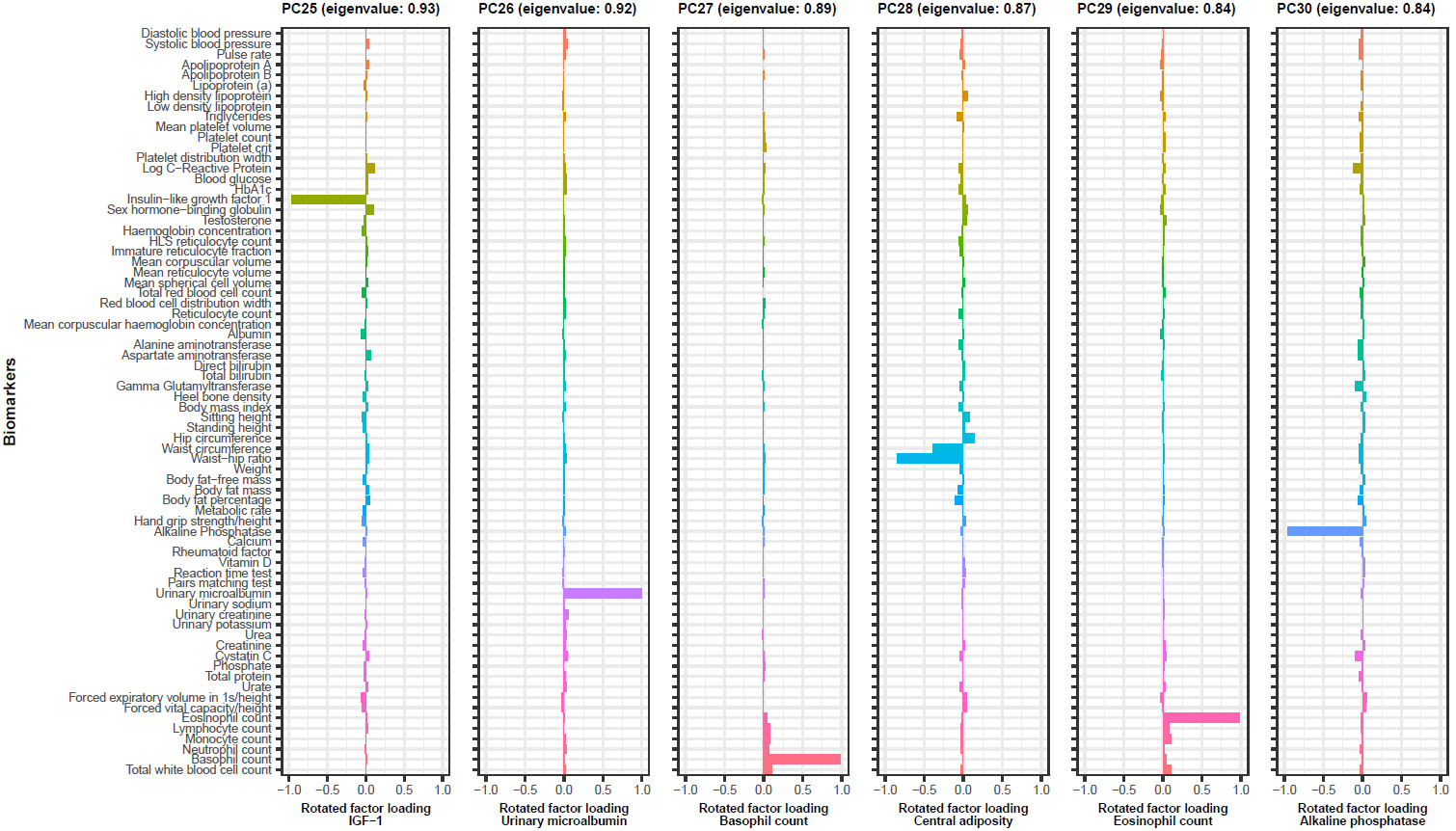

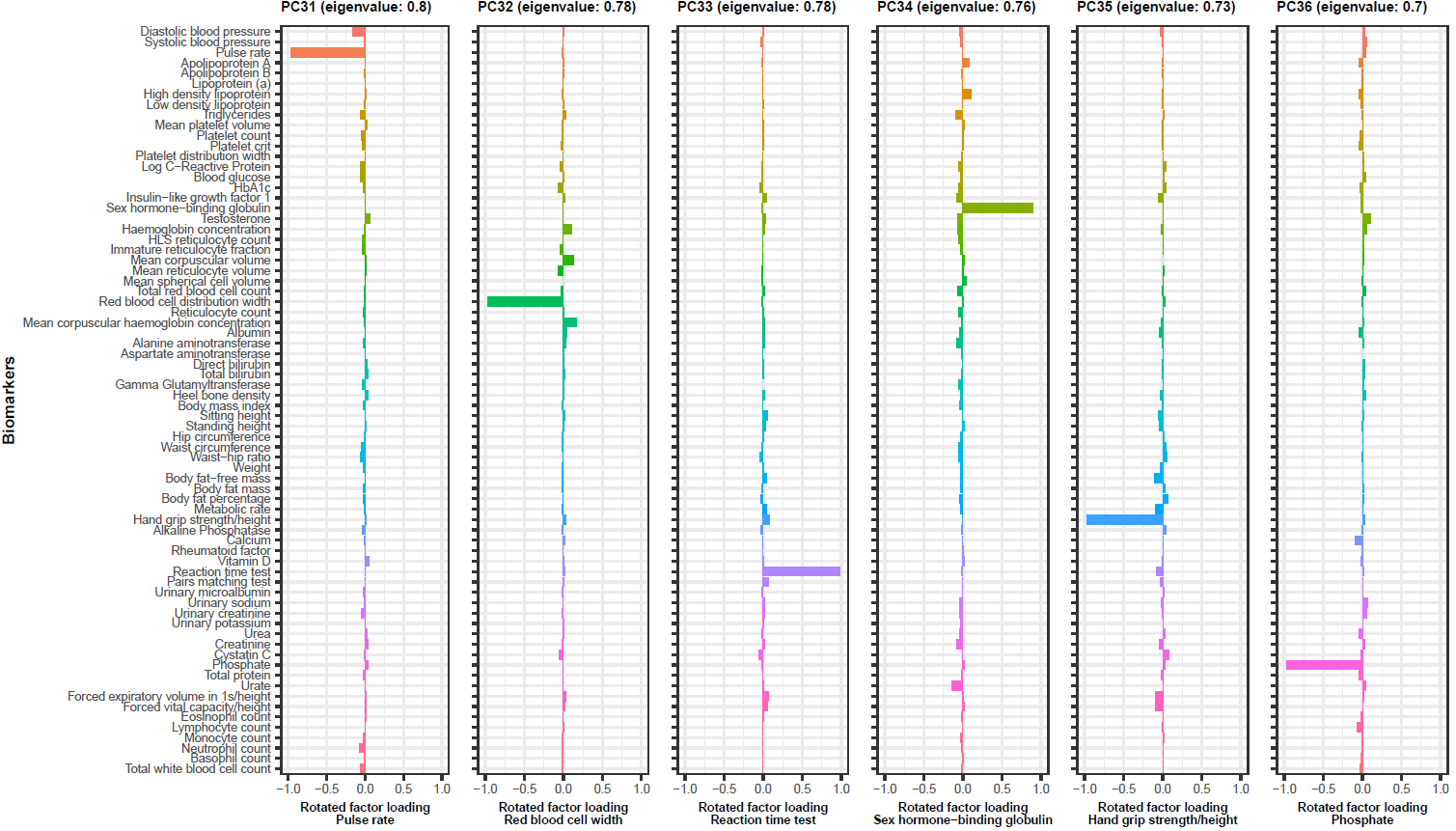

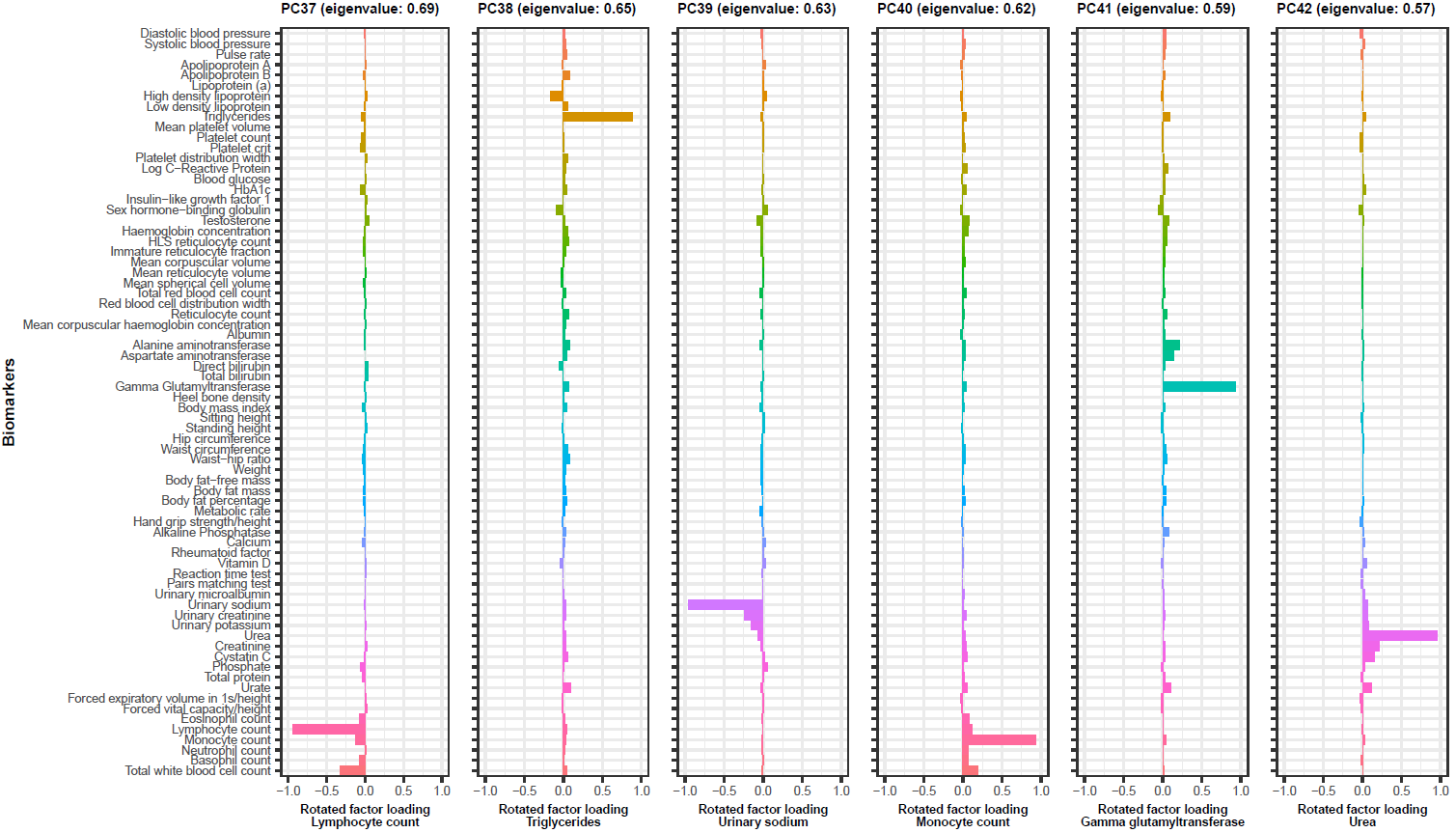

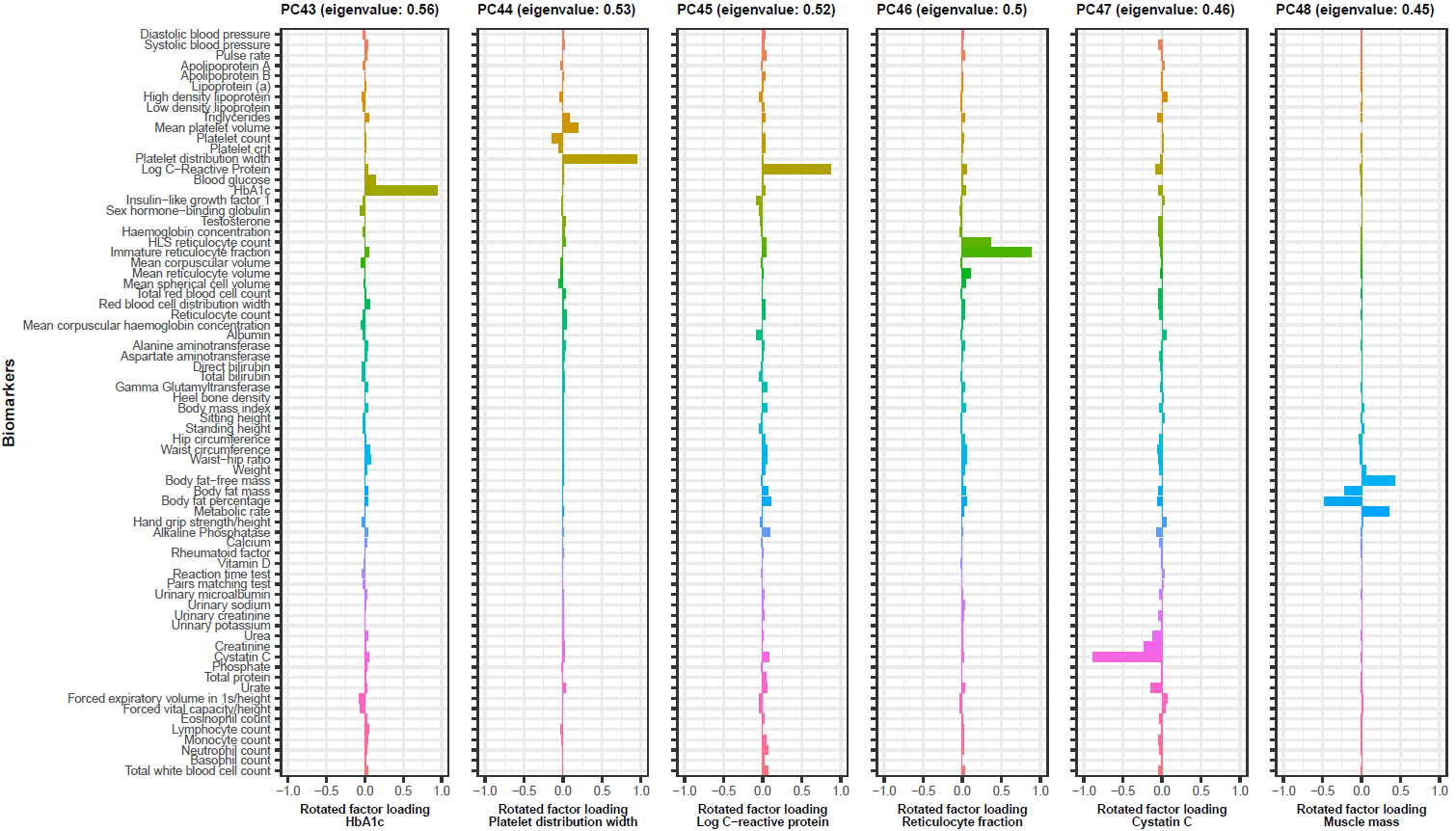

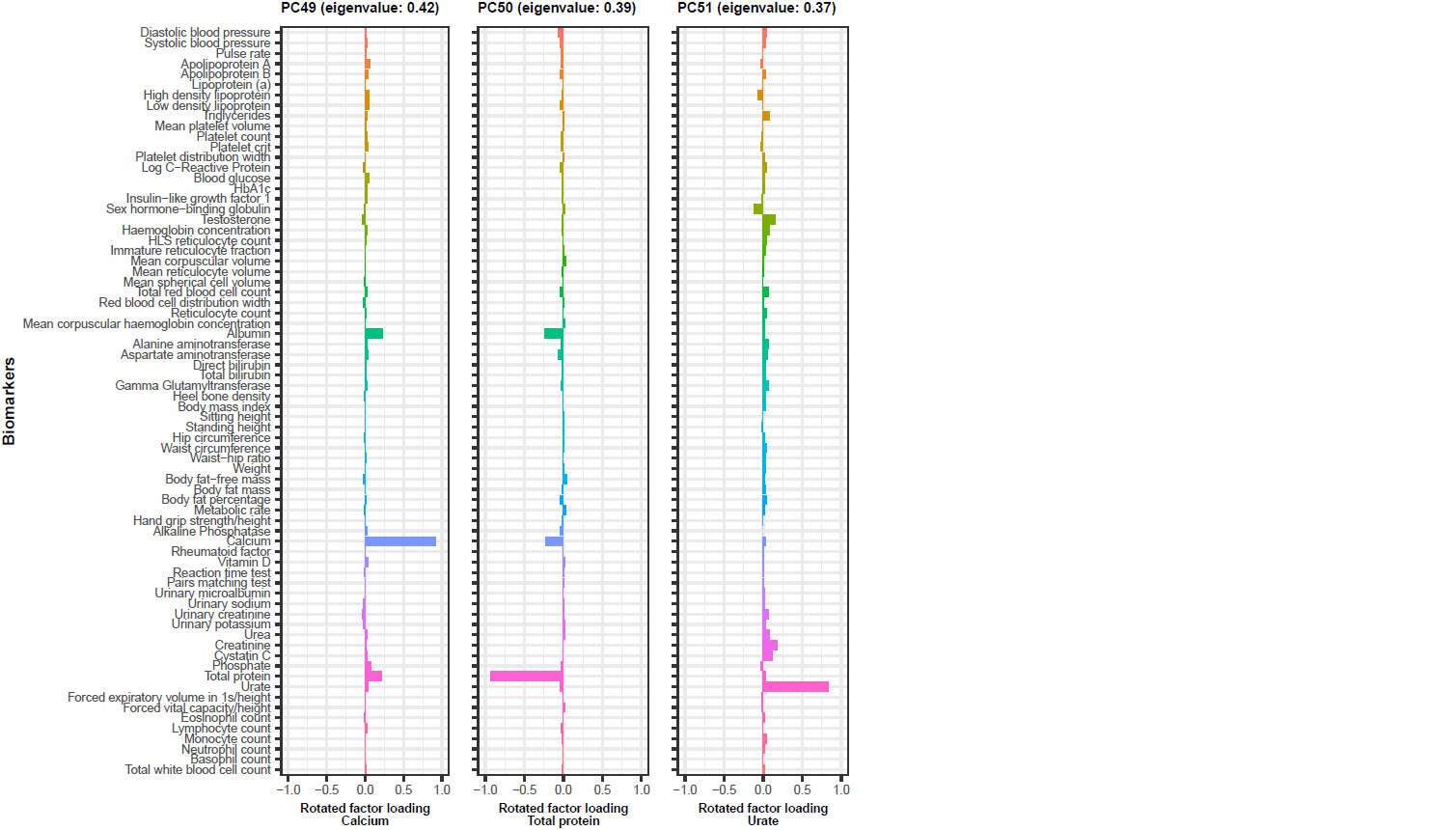


### eFigure 4: Means and standard deviations of biomarker ages by 2.5-year chronological age groups, for healthy men and healthy women

| **Healthy men** |  |  | **Healthy women** |  |
| --- | --- | --- | --- | --- |


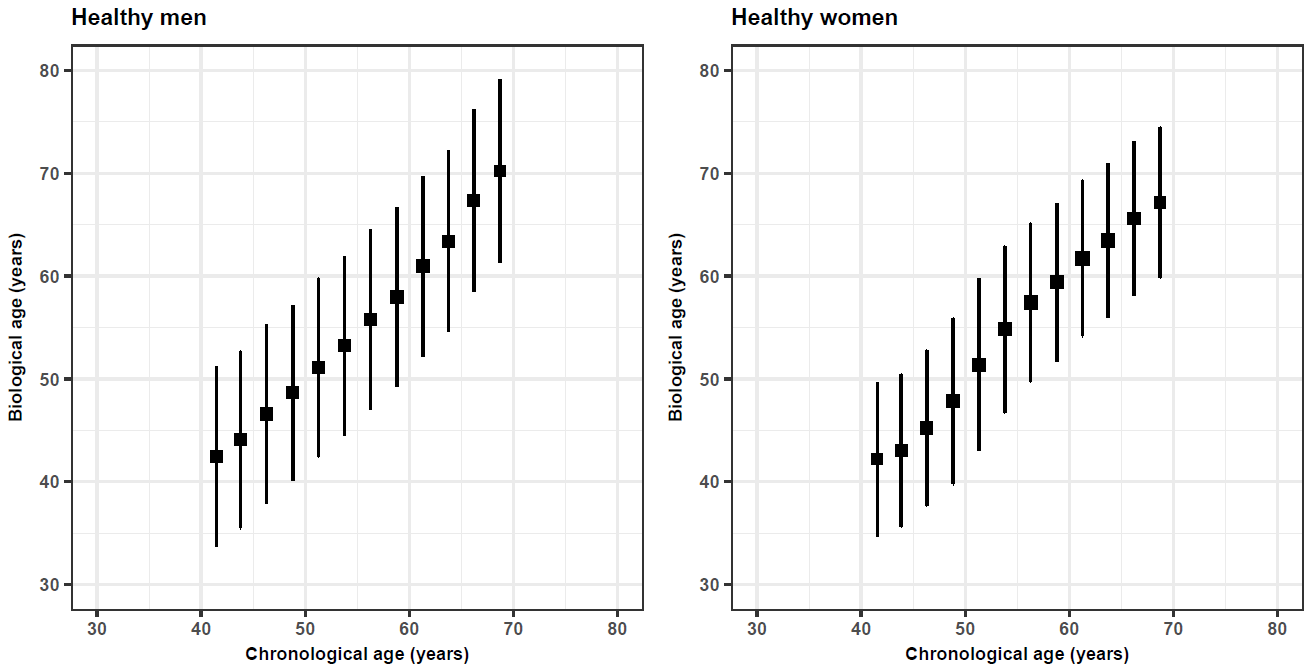


Note: These plots indicate how well biomarker ages (y-axes) are calibrated to chronological age (x-axes), and the variability (indicated by ±1 standard deviation bars) of individuals’ biomarker ages in each 2.5-year chronological age group. A perfectly calibrated biomarker age would have mean biomarker age equal to mean chronological age in each age band.

### eFigure 5: Importance of the top 15 biomarker principal components in the biomarker ages for men and women in the whole UK Biobank population


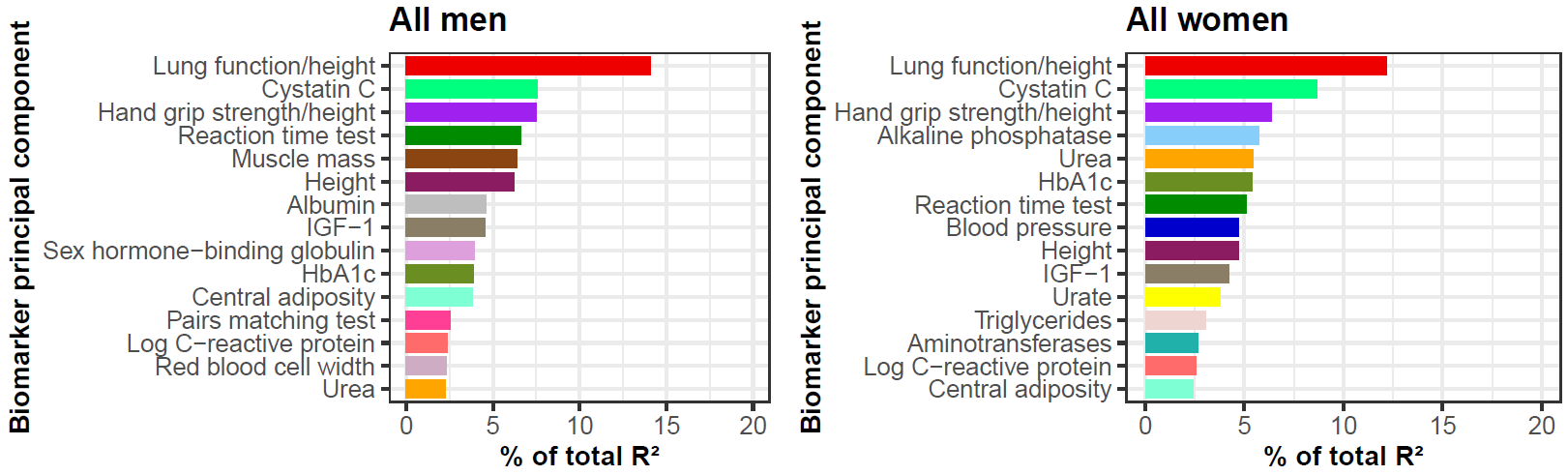


Note: The percentage of R^2^ denotes the percentage of variation in the biomarker age explained by each biomarker.

### eFigure 6: Relative contribution of biomarker ages and chronological age in explaining each health outcome, for men and women in the whole UK Biobank population


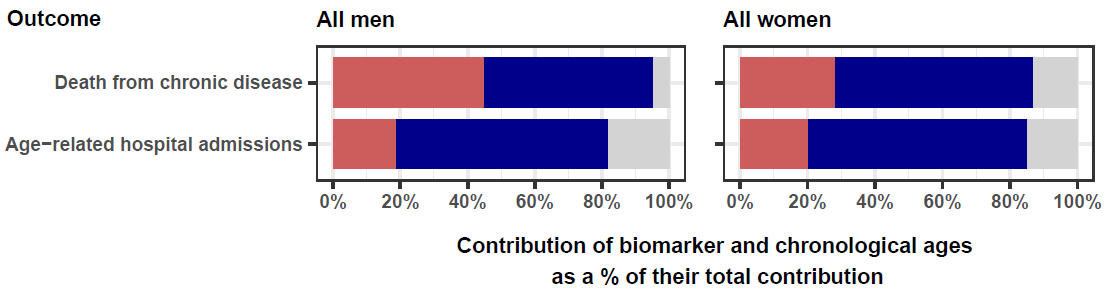

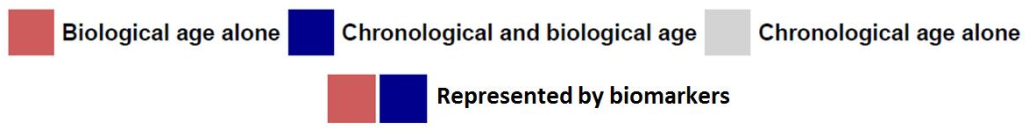

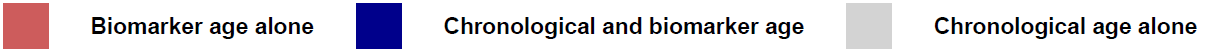


### eFigure 7: Outcome-free survival of men and women in the whole UK Biobank population for (A) mortality from chronic disease and (B) age-related hospital admissions, according to whether their biomarker age is younger, similar to or older than their chronological age

**(A) Mortality from chronic disease**


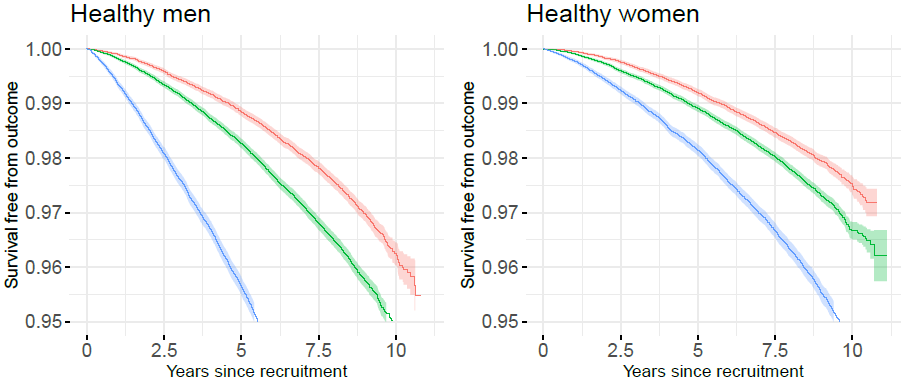


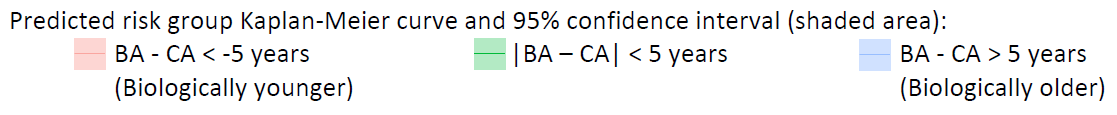
**(B) Age-related hospital admissions**


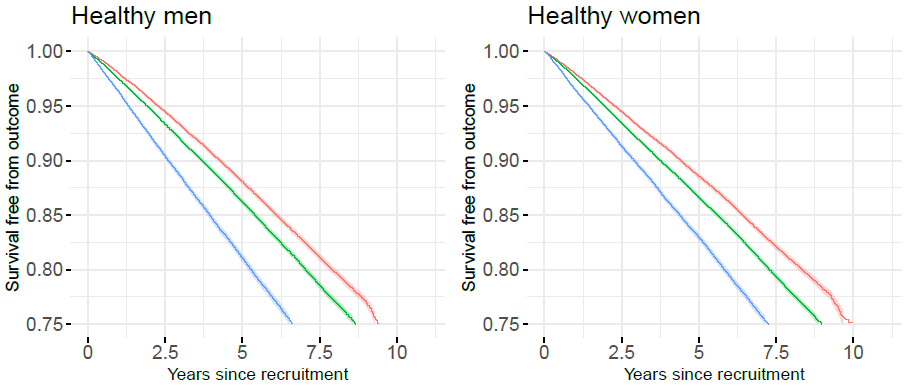


Note: BA: biomarker age; CA: chronological age.

# Supplementary References

1. Sudlow C, Gallacher J, Allen N, Beral V, Burton P, Danesh J*, et al.* UK Biobank: An Open Access Resource for Identifying the Causes of a Wide Range of Complex Diseases of Middle and Old Age. PLoS Med. 2015;**12**:e1001779. doi: 10.1371/journal.pmed.1001779

2. UK Biobank. UK Biobank. http://www.ukbiobank.ac.uk/. Accessed 1 August 2019.

3. NHS Business Services Authority. BNF Code Information. https://www.nhsbsa.nhs.uk/. Accessed 6 December 2017.

4. Tran J, Norton R, Conrad N, Rahimian F, Canoy D, Nazarzadeh M*, et al.* Patterns and temporal trends of comorbidity among adult patients with incident cardiovascular disease in the UK between 2000 and 2014: A population-based cohort study. PLoS Med. 2018;**15**:e1002513. doi: 10.1371/journal.pmed.1002513

5. Jia L, Zhang W, Chen X. Common methods of biological age estimation. Clin Interv Aging. 2017;**12**:759-772. doi: 10.2147/CIA.S134921

6. Gupta RP, Strachan DP. Ventilatory function as a predictor of mortality in lifelong non-smokers: evidence from large British cohort studies. BMJ Open. 2017;**7**:e015381. doi: 10.1136/bmjopen-2016-015381

7. Nakamura E, Miyao K. A method for identifying biomarkers of aging and constructing an index of biological age in humans. J Gerontol A Biol Sci Med Sci. 2007;**62**:1096-1105. doi: 10.1093/gerona/62.10.1096

8. UK Biobank. Companion Document to Accompany Serum Biomarker Data. http://biobank.ctsu.ox.ac.uk/crystal/crystal/docs/serum_biochemistry.pdf. Accessed 22 July 2019.

9. Yoo J, Kim Y, Cho ER, Jee SH. Biological age as a useful index to predict seventeen-year survival and mortality in Koreans. BMC Geriatr. 2017;**17**:7. doi: 10.1186/s12877-016-0407-y

10. Mamoshina P, Kochetov K, Putin E, Cortese F, Aliper A, Lee WS*, et al.* Population specific biomarkers of human aging: a big data study using South Korean, Canadian and Eastern European patient populations. J Gerontol A Biol Sci Med Sci. 2018. doi: 10.1093/gerona/gly005

11. Levine ME. Modeling the rate of senescence: can estimated biological age predict mortality more accurately than chronological age? J Gerontol A Biol Sci Med Sci. 2013;**68**:667-674. doi: 10.1093/gerona/gls233

12. Levine ME, Crimmins EM. A comparison of methods for assessing mortality risk. Am J Hum Biol. 2014;**26**:768-776. doi: 10.1002/ajhb.22595

13. Jee H, Park J. Selection of an optimal set of biomarkers and comparative analyses of biological age estimation models in Korean females. Arch Gerontol Geriatr. 2017;**70**:84-91. doi: 10.1016/j.archger.2017.01.005

14. Mitnitski A, Howlett SE, Rockwood K. Heterogeneity of Human Aging and Its Assessment. J Gerontol A Biol Sci Med Sci. 2017;**72**:877-884. doi: 10.1093/gerona/glw089

15. Gilbert T, Neuburger J, Kraindler J, Keeble E, Smith P, Ariti C*, et al.* Development and validation of a Hospital Frailty Risk Score focusing on older people in acute care settings using electronic hospital records: an observational study. The Lancet. 2018;**391**:1775-1782. doi: 10.1016/S0140-6736(18)30668-8

16. Hanlon P, Nicholl BI, Jani BD, Lee D, McQueenie R, Mair FS. Frailty and pre-frailty in middle-aged and older adults and its association with multimorbidity and mortality: a prospective analysis of 493 737 UK Biobank participants. The Lancet Public Health. 2018;**3**:e323-e332. doi: 10.1016/S2468-2667(18)30091-4

17. Vetrano DL, Palmer K, Marengoni A, Marzetti E, Lattanzio F, Roller-Wirnsberger R*, et al.* Frailty and Multimorbidity: A Systematic Review and Meta-analysis. J Gerontol A Biol Sci Med Sci. 2019;**74**:659-666. doi: 10.1093/gerona/gly110

18. Hoogendijk EO, Afilalo J, Ensrud KE, Kowal P, Onder G, Fried LP. Frailty: implications for clinical practice and public health. The Lancet. 2019;**394**:1365-1375. doi: 10.1016/S0140-6736(19)31786-6

19. Zhang W, Jia L, Cai G, Shao F, Lin H, Liu Z*, et al.* Model Construction for Biological Age Based on a Cross-Sectional Study of a Healthy Chinese Han population. Journal of Nutrition, Health & Aging. 2017;**21**:1233-1239. doi: 10.1007/s12603-017-0874-7

20. Belsky DW, Caspi A, Houts R, Cohen HJ, Corcoran DL, Danese A*, et al.* Quantification of biological aging in young adults. Proc Natl Acad Sci U S A. 2015;**112**:E4104-4110. doi: 10.1073/pnas.1506264112

21. Cho IH, Park KS, Lim CJ. An empirical comparative study on biological age estimation algorithms with an application of Work Ability Index (WAI). Mech Ageing Dev. 2010;**131**:69-78. doi: 10.1016/j.mad.2009.12.001

22. Zhong X, Lu Y, Gao Q, Nyunt MSZ, Fulop T, Monterola CP*, et al.* Estimating Biological Age in the Singapore Longitudinal Aging Study. J Gerontol A Biol Sci Med Sci. 2019. doi: 10.1093/gerona/glz146

23. Klemera P, Doubal S. A new approach to the concept and computation of biological age. Mech Ageing Dev. 2006;**127**:240-248. doi: 10.1016/j.mad.2005.10.004

24. Nakamura E, Miyao K, Ozeki T. Assessment of biological age by principal component analysis. Mech Ageing Dev. 1988;**46**:1-18. doi: 10.1016/0047-6374(88)90109-1

25. Ganna A, Ingelsson E. 5 year mortality predictors in 498,103 UK Biobank participants: a prospective population-based study. Lancet. 2015;**386**:533-540. doi: 10.1016/s0140-6736(15)60175-1

26. Liu Z, Kuo P-L, Horvath S, Crimmins E, Ferrucci L, Levine M. A new aging measure captures morbidity and mortality risk across diverse subpopulations from NHANES IV: A cohort study. PLoS Med. 2018;**15**:e1002718. doi: 10.1371/journal.pmed.1002718

27. Genizi A. Decomposition of R2 in multiple regression with correlated regressors. Statistica Sinica. 1993;**3**:407-420.

28. Groemping U. Relative Importance for Linear Regression in R: The Package relaimpo. Journal of Statistical Software. 2006;**17**:27. doi: 10.18637/jss.v017.i01

29. Grömping U. Variable importance in regression models. Wiley Interdisciplinary Reviews: Computational Statistics. 2015;**7**:137-152. doi: 10.1002/wics.1346

30. Therneau T. A Package for Survival Analysis in S. Version 2.38. https://CRAN.R-project.org/package=survival. Accessed 10 December 2019.

31. Nagelkerke NJD. A note on a general definition of the coefficient of determination. Biometrika. 1991;**78**:691-692. doi: 10.1093/biomet/78.3.691

32. Shrine N, Guyatt AL, Erzurumluoglu AM, Jackson VE, Hobbs BD, Melbourne CA*, et al.* New genetic signals for lung function highlight pathways and chronic obstructive pulmonary disease associations across multiple ancestries. Nat Genet. 2019;**51**:481-493. doi: 10.1038/s41588-018-0321-7

33. van der Laan SW, Fall T, Soumare A, Teumer A, Sedaghat S, Baumert J*, et al.* Cystatin C and Cardiovascular Disease: A Mendelian Randomization Study. J Am Coll Cardiol. 2016;**68**:934-945. doi: 10.1016/j.jacc.2016.05.092

34. Shlipak MG, Sarnak MJ, Katz R, Fried LF, Seliger SL, Newman AB*, et al.* Cystatin C and the Risk of Death and Cardiovascular Events among Elderly Persons. N Engl J Med. 2005;**352**:2049-2060. doi: 10.1056/NEJMoa043161

35. Shlipak MG, Matsushita K, Arnlov J, Inker LA, Katz R, Polkinghorne KR*, et al.* Cystatin C versus creatinine in determining risk based on kidney function. N Engl J Med. 2013;**369**:932-943. doi: 10.1056/NEJMoa1214234

36. Shipley BA, Der G, Taylor MD, Deary IJ. Cognition and all-cause mortality across the entire adult age range: health and lifestyle survey. Psychosom Med. 2006;**68**:17-24. doi: 10.1097/01.psy.0000195867.66643.0f

37. Barake M, Arabi A, Nakhoul N, El-Hajj Fuleihan G, El Ghandour S, Klibanski A*, et al.* Effects of growth hormone therapy on bone density and fracture risk in age-related osteoporosis in the absence of growth hormone deficiency: a systematic review and meta-analysis. Endocrine. 2018;**59**:39-49. doi: 10.1007/s12020-017-1440-0

38. Williams DM, Karlsson IK, Pedersen NL, Hagg S. Circulating insulin-like growth factors and Alzheimer disease: A mendelian randomization study. Neurology. 2018;**90**:e291-e297. doi: 10.1212/wnl.0000000000004854

39. Andreassen M, Raymond I, Kistorp C, Hildebrandt P, Faber J, Kristensen LO. IGF1 as predictor of all cause mortality and cardiovascular disease in an elderly population. Eur J Endocrinol. 2009;**160**:25-31. doi: 10.1530/eje-08-0452

40. Tumati S, Burger H, Martens S, van der Schouw YT, Aleman A. Association between Cognition and Serum Insulin-Like Growth Factor-1 in Middle-Aged & Older Men: An 8 Year Follow-Up Study. PLoS One. 2016;**11**:e0154450. doi: 10.1371/journal.pone.0154450

41. Farmer RE, Mathur R, Schmidt AF, Bhaskaran K, Fatemifar G, Eastwood SV*, et al.* Associations Between Measures of Sarcopenic Obesity and Risk of Cardiovascular Disease and Mortality: A Cohort Study and Mendelian Randomization Analysis Using the UK Biobank. J Am Heart Assoc. 2019;**8**:e011638. doi: 10.1161/jaha.118.011638

42. Ho FKW, Celis-Morales CA, Petermann-Rocha F, Sillars A, Welsh P, Welsh C*, et al.* The association of grip strength with health outcomes does not differ if grip strength is used in absolute or relative terms: a prospective cohort study. Age Ageing. 2019. doi: 10.1093/ageing/afz068

43. Ettehad D, Emdin CA, Kiran A, Anderson SG, Callender T, Emberson J*, et al.* Blood pressure lowering for prevention of cardiovascular disease and death: a systematic review and meta-analysis. The Lancet. 2016;**387**:957-967. doi: 10.1016/S0140-6736(15)01225-8

44. Aikens RC, Zhao W, Saleheen D, Reilly MP, Epstein SE, Tikkanen E*, et al.* Systolic Blood Pressure and Risk of Type 2 Diabetes: A Mendelian Randomization Study. Diabetes. 2017;**66**:543. doi: 10.2337/db16-0868

45. Østergaard SD, Mukherjee S, Sharp SJ, Proitsi P, Lotta LA, Day F*, et al.* Associations between Potentially Modifiable Risk Factors and Alzheimer Disease: A Mendelian Randomization Study. PLoS Med. 2015;**12**:e1001841. doi: 10.1371/journal.pmed.1001841

46. Prospective Studies Collaboration. Age-specific relevance of usual blood pressure to vascular mortality: a meta-analysis of individual data for one million adults in 61 prospective studies. The Lancet. 2002;**360**:1903-1913. doi: 10.1016/S0140-6736(02)11911-8

47. Danesh J, Collins R, Appleby P, Peto R. Association of Fibrinogen, C-reactive Protein, Albumin, or Leukocyte Count With Coronary Heart DiseaseMeta-analyses of Prospective Studies. JAMA. 1998;**279**:1477-1482. doi: 10.1001/jama.279.18.1477

48. Perry JRB, Weedon MN, Langenberg C, Jackson AU, Lyssenko V, Sparsø T*, et al.* Genetic evidence that raised sex hormone binding globulin (SHBG) levels reduce the risk of type 2 diabetes. Hum Mol Genet. 2009;**19**:535-544. doi: 10.1093/hmg/ddp522

49. Wang Q, Kangas AJ, Soininen P, Tiainen M, Tynkkynen T, Puukka K*, et al.* Sex hormone-binding globulin associations with circulating lipids and metabolites and the risk for type 2 diabetes: observational and causal effect estimates. Int J Epidemiol. 2015;**44**:623-637. doi: 10.1093/ije/dyv093

50. Oppert JM, Charles MA, Thibult N, Guy-Grand B, Eschwege E, Ducimetiere P. Anthropometric estimates of muscle and fat mass in relation to cardiac and cancer mortality in men: the Paris Prospective Study. Am J Clin Nutr. 2002;**75**:1107-1113. doi: 10.1093/ajcn/75.6.1107

51. Janssen I, Heymsfield SB, Ross R. Low relative skeletal muscle mass (sarcopenia) in older persons is associated with functional impairment and physical disability. J Am Geriatr Soc. 2002;**50**:889-896. doi: 10.1046/j.1532-5415.2002.50216.x

52. Lai FY, Nath M, Hamby SE, Thompson JR, Nelson CP, Samani NJ. Adult height and risk of 50 diseases: a combined epidemiological and genetic analysis. BMC Med. 2018;**16**:187. doi: 10.1186/s12916-018-1175-7

53. Emerging Risk Factors C. Adult height and the risk of cause-specific death and vascular morbidity in 1 million people: individual participant meta-analysis. Int J Epidemiol. 2012;**41**:1419-1433. doi: 10.1093/ije/dys086

54. Cholesterol Treatment Trialists Collaborators, Mihaylova B, Emberson J, Blackwell L, Keech A, Simes J*, et al.* The effects of lowering LDL cholesterol with statin therapy in people at low risk of vascular disease: meta-analysis of individual data from 27 randomised trials. Lancet. 2012;**380**:581-590. doi: 10.1016/S0140-6736(12)60367-5

55. Holmes MV, Asselbergs FW, Palmer TM, Drenos F, Lanktree MB, Nelson CP*, et al.* Mendelian randomization of blood lipids for coronary heart disease. Eur Heart J. 2015;**36**:539-550. doi: 10.1093/eurheartj/eht571

56. Sniderman AD, Williams K, Contois JH, Monroe HM, McQueen MJ, de Graaf J*, et al.* A meta-analysis of low-density lipoprotein cholesterol, non-high-density lipoprotein cholesterol, and apolipoprotein B as markers of cardiovascular risk. Circ Cardiovasc Qual Outcomes. 2011;**4**:337-345. doi: 10.1161/circoutcomes.110.959247

57. De Silva NMG, Borges MC, Hingorani A, Engmann J, Shah T, Zhang X*, et al.* Liver Function and Risk of Type 2 Diabetes: Bidirectional Mendelian Randomization Study. Diabetes. 2019:db181048. doi: 10.2337/db18-1048

58. Liu J, Au Yeung SL, Lin SL, Leung GM, Schooling CM. Liver Enzymes and Risk of Ischemic Heart Disease and Type 2 Diabetes Mellitus: A Mendelian Randomization Study. Sci Rep. 2016;**6**:38813. doi: 10.1038/srep38813

59. Hao H, Chen L, Huang D, Ge J, Qiu Y, Hao L. Meta-analysis of alkaline phosphatase and prognosis for osteosarcoma. Eur J Cancer Care (Engl). 2017;**26**. doi: 10.1111/ecc.12536

60. Kunutsor SK, Bakker SJL, Kootstra-Ros JE, Gansevoort RT, Gregson J, Dullaart RPF. Serum Alkaline Phosphatase and Risk of Incident Cardiovascular Disease: Interrelationship with High Sensitivity C-Reactive Protein. PLoS One. 2015;**10**:e0132822. doi: 10.1371/journal.pone.0132822

61. Griffin SJ, Leaver JK, Irving GJ. Impact of metformin on cardiovascular disease: a meta-analysis of randomised trials among people with type 2 diabetes. Diabetologia. 2017;**60**:1620-1629. doi: 10.1007/s00125-017-4337-9

62. Au Yeung SL, Luo S, Schooling CM. The Impact of Glycated Hemoglobin (HbA1c) on Cardiovascular Disease Risk: A Mendelian Randomization Study Using UK Biobank. Diabetes Care. 2018:dc180289. doi: 10.2337/dc18-0289

63. Campbell JM, Bellman SM, Stephenson MD, Lisy K. Metformin reduces all-cause mortality and diseases of ageing independent of its effect on diabetes control: A systematic review and meta-analysis. Ageing Res Rev. 2017;**40**:31-44. doi: 10.1016/j.arr.2017.08.003

64. Jiang H, Li J, Yu K, Yang H, Min X, Chen H*, et al.* Associations of estimated glomerular filtration rate and blood urea nitrogen with incident coronary heart disease: the Dongfeng-Tongji Cohort Study. Sci Rep. 2017;**7**:9987. doi: 10.1038/s41598-017-09591-6

65. Collins GS, Reitsma JB, Altman DG, Moons KGM. Transparent Reporting of a multivariable prediction model for Individual Prognosis Or Diagnosis (TRIPOD): The TRIPOD StatementThe TRIPOD Statement. Ann Intern Med. 2015;**162**:55-63. doi: 10.7326/M14-0697
